# Anhydrobiosis and Freezing-Tolerance: Adaptations That Facilitate the Establishment of *Panagrolaimus* Nematodes in Polar Habitats

**DOI:** 10.1371/journal.pone.0116084

**Published:** 2015-03-06

**Authors:** Lorraine M. McGill, Adam J. Shannon, Davide Pisani, Marie-Anne Félix, Hans Ramløv, Ilona Dix, David A. Wharton, Ann M. Burnell

**Affiliations:** 1 Department of Biology, Maynooth University, Maynooth, Co Kildare, Ireland; 2 Technology Sciences Group Europe LLP, Concordia House, St James Business Park, Knaresborough, North Yorkshire, HG5 8QB, United Kingdom; 3 School of Biological Sciences and School of Earth Sciences, University of Bristol, Woodland Road, BS8 1UG, Bristol, United Kingdom; 4 Institute of Biology of the Ecole Normale Supérieure, 46 rue d’Ulm, 75230 Paris cedex 05, France; 5 Department of Science, Systems and Models, Roskilde University, Universitetsvej 1, P.O.Box 260, DK-4000 Roskilde, Denmark; 6 Department of Zoology, University of Otago, PO Box 56, Dunedin 9054, New Zealand; Chinese Academy of Sciences, CHINA

## Abstract

Anhydrobiotic animals can survive the loss of both free and bound water from their cells. While in this state they are also resistant to freezing. This physiology adapts anhydrobiotes to harsh environments and it aids their dispersal. *Panagrolaimus davidi*, a bacterial feeding anhydrobiotic nematode isolated from Ross Island Antarctica, can survive intracellular ice formation when fully hydrated. A capacity to survive freezing while fully hydrated has also been observed in some other Antarctic nematodes. We experimentally determined the anhydrobiotic and freezing-tolerance phenotypes of 24 *Panagrolaimus* strains from tropical, temperate, continental and polar habitats and we analysed their phylogenetic relationships. We found that several other *Panagrolaimus* isolates can also survive freezing when fully hydrated and that tissue extracts from these freezing-tolerant nematodes can inhibit the growth of ice crystals. We show that *P. davidi* belongs to a clade of anhydrobiotic and freezing-tolerant panagrolaimids containing strains from temperate and continental regions and that *P. superbus*, an early colonizer at Surtsey island, Iceland after its volcanic formation, is closely related to a species from Pennsylvania, USA. Ancestral state reconstructions show that anhydrobiosis evolved deep in the phylogeny of *Panagrolaimus*. The early-diverging *Panagrolaimus* lineages are strongly anhydrobiotic but weakly freezing-tolerant, suggesting that freezing tolerance is most likely a derived trait. The common ancestors of the *davidi* and the *superbus* clades were anhydrobiotic and also possessed robust freezing tolerance, along with a capacity to inhibit the growth and recrystallization of ice crystals. Unlike other endemic Antarctic nematodes, the life history traits of *P. davidi* do not show evidence of an evolved response to polar conditions. Thus we suggest that the colonization of Antarctica by *P. davidi* and of Surtsey by *P. superbus* may be examples of recent “ecological fitting” of freezing-tolerant anhydrobiotic propagules to the respective abiotic conditions in Ross Island and Surtsey.

## Introduction

Anhydrobiosis is a term used to describe the prolonged, reversible, ametabolic state that some organisms can attain in order to survive conditions of extreme desiccation [1]. Anhydrobiotes are capable of losing between 95–99% of their body water during this process [1,2]. This physiology is adapted to drought-prone environments, and also aids the dispersal of desiccated anhydrobiotes [3]. Taxa that can enter anhydrobiosis at all life stages include some bacteria, lichens, mosses, ferns, angiosperm “resurrection plants”, free-living nematodes, rotifers and tardigrades [4,5,6]. In other taxa anhydrobiosis is stage-specific: spores of some bacteria and fungi; embryonic cysts of brine shrimps and some other microcrustaceans; larvae of some parasitic nematodes; larvae of the chironomid insect *Polypedilum vanderplanki* and many plant seeds and pollens [4,6,7]. During anhydrobiosis metabolism and life processes come to a halt, but they resume again upon rehydration. While in an anhydrobiotic state organisms are also resistant to freezing, because they lack the water necessary to form tissue-damaging ice crystals [8,9]. Anhydrobiotes synthesise a variety of molecules to protect their macromolecules and cellular structures during desiccation [6,10,11,12]. These include water replacement molecules such as trehalose or other sugars; antioxidants; molecular chaperones; LEA (= “late embryogenesis abundant) proteins, which stabilize cellular proteins and prevent their aggregation; and desaturases which adjust membrane fluidity. Most anhydrobiotic organisms need to be dehydrated slowly to allow time for these metabolic adjustments to occur, but some “fast-dehydration strategist” nematodes [13,14] and mosses [15] can survive rapid dehydration.

A link between desiccation tolerance and cold tolerance is evident in organisms capable of undergoing cryoprotective dehydration. When soil freezes, some freeze-avoiding invertebrates with high integumental water permeability dehydrate until the melting point of their body fluids equals that of the frozen soil [16]. Some Collembola from polar and subpolar regions are examples of such organisms [17]. To protect their tissues from the effects of dehydration these micro-invertebrates synthesize osmolytes, molecular chaperones and antioxidants [18]. Freeze-avoiding fish and insects rely on antifreeze ice-active proteins (IAPs) to lower the freezing temperature of their supercooled body fluids to a point below the temperature of the ambient environment [19,20]. IAPs are very diverse in sequence and structure [21,22,23]. They function by binding to ice crystal surfaces and inhibiting their growth [21]. Antifreeze IAPs inhibit the growth of ice crystals over a range of temperatures in supercooled body fluids without altering the melting point of ice [20,24], an activity known as thermal hysteresis (TH). The temperature at which the ice crystal eventually grows is called the thermal hysteresis freezing point (THP). Within the TH gap ice crystals do not grow or melt [24]. The TH gap (the difference between the THP and the melting point) of fish IAP solutions is in the range 0.5–1.0°C [25], whereas insect IAPs have more potent TH activity, being capable of depressing the freezing point of tissue fluids by 3.5–4°C [22,23,26]. Insect IAPs, in synergy with the synthesis of polyols such as glycerol and the removal/inhibition of ice nucleating agents, can prevent freezing to -30°C [20]. IAPs isolated from plants and microorganisms generally have low TH activity, their main function being to inhibit the growth and recrystallization of ice crystals and its associated tissue damage [27,28]. *Panagrolaimus davidi* from Ross Island Antarctica is the best characterized example of a nematode that is capable of surviving intracellular ice formation when fully hydrated [29] and this capacity is associated with the synthesis of an IAP which inhibits the growth of ice crystals but lacks TH activity [30].

Panagrolaimid nematodes are bacterivores, occupying niches ranging from polar, temperate and semi-arid soils to terrestrial mosses [14]. They have been isolated from several sites in Antarctica, where they are abundant in the bacteria-rich soil associated with bird nesting sites [31] and *Panagrolaimus superbus* was isolated in 1981 from a gull’s nest in Surtsey [32], an icelandic island formed during 1963 to 1967 by volcanic eruptions [33]. The occurrence of freezing-tolerant phenotypes in panagrolaimids other than *P. davidi* has not been investigated previously. In the present study we experimentally determined the anhydrobiotic and freezing tolerance phenotypes of 24 *Panagrolaimus* strains from tropical, temperate, continental and polar habitats and analysed their phylogenetic relationships. We found that several other *Panagrolaimus* isolates, in addition to *P. davidi* from Antartica, are able to survive freezing when fully hydrated and that tissue extracts from these freezing-tolerant nematodes can inhibit the growth of ice crystals. These isolates include *P. superbus* from Surtsey, Iceland, along with several anhydrobiotic strains from temperate regions. Using this dataset we addressed several questions concerning the biology and evolution of freezing and desiccation tolerance in panagrolaimid nematodes:

1. Is freezing tolerance in *Panagrolaimus* associated with TH effects?
2. Did the frozen nematodes undergo cryoprotective dehydration?
3. Are the freezing-tolerant and anhydrobiotic isolates of *Panagrolaimus* closely related phylogenetically, or do these phenotypes represent instances of convergent evolution?
4. Does the pattern of association between freezing-tolerance and desiccation-tolerance phenotypes in individual isolates across the phylogeny show evidence of correlated evolution?
5. Did the evolution of freezing tolerance in *P. davidi* predate its dispersal to Antarctica?

We found that tissue extracts from freezing-tolerant nematodes lack TH activity and that photomicrographs of frozen nematodes did not show evidence of cryoprotective dehydration. Phylogenetic signal for both the anhydrobiosis and the freezing-tolerance phenotypes was high—an indication that, with regard to these traits, related panagrolaimids resemble each other more closely than they resemble randomly sampled strains from the phylogeny [34]. Ancestral trait reconstructions indicate that the early-diverging lineages in this *Panagrolaimus* phylogeny were strongly anhydrobiotic but weakly freezing tolerant. These reconstructions also show that prior to the radiation of the *P. davidi* clade and the dispersal of *P. davidi* to Antarctica the common ancestor of this clade already possessed a potent freezing-tolerant phenotype, along with a capacity to inhibit the growth and recrystallization of ice.

## Materials and Methods

### Source and Culturing of *Panagrolaimus* Isolates

The sources and geographic origins of the 24 *Panagrolaimus* isolates are listed in S1 Table. The nematodes were cultured at 20°C in the dark on nematode growth medium (NGM) plates [35] containing a lawn of streptomycin-resistant *Escherichia coli* strain HB101 obtained from the *Caenorhabditis* Genetics Center (http://www.cbs.umn.edu/cgc). The NGM was supplemented with streptomycin sulfate (30 μg mL^-1^). Twelve new *Panagrolaimus* strains were isolated using the agar plate method described by Barrière and Félix [36]. Mixed-stage nematodes (i.e. containing a mixture of larvae and adult males and females) were harvested from the NGM plates using distilled water. The nematode suspension was transferred to 50 ml Falcon tubes and the nematodes were separated from their *E*. *coli* food source by sedimentation at room temperature (or at 10°C for the cold acclimated nematodes). The supernatant containing the *E*. *coli* was removed and fresh distilled water was added. This settling process was repeated a total of three times.

### Desiccation Tolerance

Desiccation tolerance was determined using mixed-stage nematode populations. A 1 mL suspension of freshly-harvested nematodes in distilled water (concentration 2,000 nematodes mL^-1^) was vacuum filtered onto a 2.5 cm Supor-450 filter (Merk Millipore, Billerica, Ma, USA). The filters were then transferred to 3 cm Petri dishes without lids and placed in a 10.0 L desiccation chamber containing approximately 300 mL of saturated potassium dichromate (K_2_Cr_2_O_7_) which maintained a relative humidity (RH) of 98% [37]. Following different preconditioning treatments (0 h, 24 h, 48 h, 72 h or 96 h at 98% RH) the nematode dishes were placed in sealed plastic boxes containing freshly activated silica gel for 48 h. Then the desiccated nematodes were rehydrated by adding 1 mL of distilled water to each Petri dish. The dishes were incubated with shaking at 50 rpm, for 24 h at 20°C and post-rehydration survival was assessed by microscopic observation of nematode motility. Three biological replicates (i.e. separate Supor filters) were prepared for each treatment and nematode survival counts were obtained for four separate aliquots from each filter. For each aliquot a minimum of 80 nematodes was observed.

### Freezing Tolerance

One mL suspensions of freshly-harvested mixed-stage nematodes (2,000 nematodes mL^-1^ in distilled water) were transferred to 1.5 mL Eppendorf tubes and placed in a 935 cm^3^ (volume) polystyrene box in a -80°C freezer which provided a cooling rate of 3.02°C min^-1^. A single freezing exotherm was detected at -4.6°C (S1 Fig.). The duration of this exotherm was 550 s and during the first 440 s of the exotherm the cooling rate decreased to 0.02°C min^-1^, thereafter the cooling rate increased rapidly towards its previous rate of ~ 3.02°C min^-1^. The cooling rate was measured using a National Semiconductor/Texas Instruments LM35A Precision Centrigade Temperature Sensor, range +150°C to -55°C (Dallas, Tx, USA). A customized LabVIEW (www.ni.com/labview/) data acquisition program interfaced with the thermocouple output through a National Instruments AT-MIO-16E-IO data acquisition board (www.ni.com). The nematodes were left in the -80°C freezer for 24 h. Then the 1 mL aliqots of frozen nematodes were thawed rapidly at 30°C, transferred to a 3 cm Petri dish and allowed to recover at 20°C for 24 h, with shaking at 50 rpm. Freezing survival was then assessed by microscopic observation of nematode movement. Four biological replicates were prepared for each experiment and nematode survival counts were obtained for four separate aliquots from each biological replicate. For each aliquot a minimum of 80 nematodes was observed.

A pilot experiment was set up to investigate the effect of cold acclimation on the freezing tolerance of *P. superbus* (S2 Fig.). The nematodes were grown at 20°C on NGM agar plates containing a lawn of *E*. *coli* until a mixed stage population had developed (~10 days). The nematodes were then acclimated (a) on the NGM agar plates at 4°C or 10°C for 4 or 10 days or (b) in 1 mL aliqots (2,000 nematodes mL^-1^ in distilled water in a 1.5 mL Eppendorf tube) at 4°C or 10°C for 6 h. Following acclimation the nematodes were frozen as described above. Twenty-four hours later the 1 mL aliqots of nematodes were thawed and their survival assessed after a 24 h recovery period at 20°C. Based on the data from the pilot experiment the assays used to assess freezing-tolerance phenotypes ulilized nematodes grown on NGM plates at 20°C (controls) or nematodes which had been acclimated on NGM plates at 10°C for 10 days (cold acclimated) prior to freezing.

### Preparation of *Panagrolaimus* Tissue Extracts for Ice Growth and Thermal Hysteresis Measurements

Mixed-stage nematodes from twenty 9 cm NGM plates were harvested as described in the main text. The cleaned nematodes were resuspended in S buffer [0.1 M NaCl and 0.05 M potassium phosphate (pH 6.0) [35], and centrifuged at 4,000g in a Hermle Z 020060A bench centrifuge. The resulting *ca*. 500 μL pellet of packed nematodes was resuspended in 0.5x volume of ice cold S buffer and the nematode suspension was dripped into liquid nitrogen in an autoclaved mortar and ground to a fine powder using an autoclaved pestle. The extract was transferred to a sterile 1.5 ml Eppendorf tube containing 0.25 g of ice-cold glass beads (<106 microns, Cat. No. G 8893, Sigma-Aldrich, St. Louis, Mo, USA). The sample was then homogenised using a Mini-beadbeater (Biospec Products, Bartlesville, Ok, USA) on ice for three 30 sec pulses at high speed, with cooling on ice for 1 min between each pulse. The extract was then centrifuged at 17,700g at 4°C for ten min in an Eppendorf 5417R centrifuge and the supernatant solution was retained. The protein concentration of the supernatant was determined using a BCA (bicinchoninic acid) protein assay kit (Novagen, Merk Millipore, Billerica, Ma, USA) and the supernatant was aliquotted and stored at -80°C until used.

### Ice-Crystal Growth Assays and Thermal Hysteresis Measurements

The ability of *Panagrolaimus* tissue extracts to inhibit the growth of a single seed ice crystal was determined using a freezing-point nanolitre osmometer (Otago Osmometers, Dunedin, NZ) following the protocols of Wharton *et al*. [30] and Ramløv *et al*. [38]. The protein concentration of the extracts was adjusted to 2 μg μL^-1^ with S buffer for use in these assays. The freezing point osmometer comprises a Peltier temperature-controlling device with an integrated microscope cryostage that was placed onto the viewing stage of an Olympus BX51 microscope. The cryostage was maintained at 0°C to load the samples. A drop of oil (Cargille’s A: Cargille Laboratories, Cedar Grove, NJ, USA) was placed into a micro well in an aluminium sample holder on the cryostage and the tissue extract (*ca*. 10 nL) was inserted into the oil drop using an elongated microcapillary tube. The extract was frozen by rapidly cooling the stage to -20°C. The cryostage temperature was increased until the ice began to melt, then the temperature was raised very slowly (0.01°C min^-1^) until the melting temperature (the temperature at which the last tiny ice crystal melted) was reached. The temperature was then decreased to refreeze the sample and it was increased again to melt the sample back to a single small ice crystal. The temperature was lowered very slowly (~ 0.01°C min^-1^) until discernable growth of the ice crystal occurred. The temperature at which ice growth recommences corresponds to the hysteresis freezing point. Thermal hysteresis (TH) was calculated as the difference between the melting temperatures of the tissue extract and its hysteresis freezing point. The morphology of the ice crystals upon growth at the hysteresis freezing point was noted and the crystals were photographed using the Olympus BX51 imaging system. TH measurements were obtained for tissue extracts from two *Panagrolaimus* strains prepared using three different buffer systems: (a) S buffer [35]; (b) 25 mM Tris HCl, pH 8.6 and (c) 0.1 M NH_4_HCO_3_ pH 7.9] (Table 1).

**Table 1:** Thermal hysteresis measurements for tissue extracts from two *Panagrolaimus* strains determined using three buffer systems.

	Thermal Hysteresis (°C)*		
	25 mM Tris HCl, pH 8.6	S buffer pH 6.0^†^	0.1 M NH_4_HCO_3_, pH 7.9
*Panagrolaimus* sp. PS1159	0.01±0	0.01±0	0.02±0
*Panagrolaimus* sp. SN103	0.02±0	0.03±0.01	0.03±0.01
Buffer control	0.02±0.01	0.03±0.01	0.02±0.01

* mean ± standard error, N = 3

^†^ 0.1 M NaCl, 0.05 M potassium phosphate, pH 6.0 [35].

Recrystallization inhibition was tested using a splat freezing assay as described by Wharton *et al*. [30]. Ten μL of each test sample was dropped onto an aluminium block that had been cooled to -78°C by dry ice. A portion of the resulting thin layer of polycrystalline ice was transferred to a microscope cold stage and the temperature was raised to -8°C (ice recrystallization is more pronounced at high sub-zero temperatures because smaller ice crystals, which have higher melting temperatures, tend to melt and reanneal with larger crystals). The ice crystals were allowed to anneal for 30 min at -8°C and were then photographed between crossed Polaroid filters. The maximum diameter of the ten most prominent ice crystals in each photograph was measured using the public domain Image J program (http://rsbweb.nih.gov/ij/).

### Microscopy of Thawed Nematodes

One mL suspensions of freshly-harvested mixed-stage nematodes (2,000 nematodes mL^-1^ in distilled water) were frozen as described above. After 24 h the frozen contents of a single Eppendorf tube were transferred to a Petri dish. When the ice began to melt emerging nematodes were immediately transferred, using an aspirator, to an air-dried 2% agarose pad on a cover slip. The cover slip was placed on the stage of an Olympus SZX16 microscope and the nematodes were photographed immediately. The agarose pads, which help to immobilize the nematodes, were prepared as described by Fire [39].

### Principal Component Analysis

Principal component analysis was performed on the proportional desiccation and freezing data (five desiccation treatments and two freezing treatments-S2 Table), using the princomp function in R. The multivariate normality of the untransformed data was first confirmed (*P*<0.01) using the mshapiro.test function (Shapiro-Wilk test) in the R software package mvnormtest. Prior to PCA the proportional data were scaled by subtracting the mean from the standard deviation of each sample.

### Phylogenetic and Evolutionary Analyses

#### (a) Phylogenetic reconstruction (tree building)

Nematode DNA was extracted using a DNeasy Blood and Tissue extraction Kit (Qiagen, N.V. Netherlands). The nematodes were harvested as described above and a 100–200 μL packed nematode pellet was ground in 200 μL of nematode lysis buffer [20 mM Tris HCl pH 7.5, 50 mM EDTA, 200 mM NaCl, 0.5% (w/v) SDS] in an autoclaved mortar and pestle using liquid nitrogen. The remainder of the extraction protocol followed the manufacturer’s instructions for animal tissues, including the RNase A digestion step. The 28S rDNA D3 expansion region was PCR amplified using the primers D3A 5’-GACCCGTCTTGAAACACGGA-3’ and D3B 5’ TCGGAAGGAACCAGCTACTA3’ [40] as previously described [14]. PCR amplicons were purified using a QIAquick PCR purification kit (Qiagen), cloned into the pJET1.2/blunt vector (Fermentas, Thermo Scientific, Waltham, Ma, USA) and transformed into *E*. *coli* TOP10 cells (Invitrogen), according to the manufacturers’ instructions. The plasmids were purified from *E*. *coli* cultures using a QIAprep Spin Miniprep kit (Qiagen) and the purified plasmids were sequenced by LGC Genomics (Berlin, Germany). For each nematode strain the inserts from at least two recombinant plasmids were sequenced in the forward and reverse directions. The GenBank accession numbers of these D3 sequences are presented in S3 Table.

Multiple sequence alignments were created using ClustalW2 [41] as implemented in Mega 5.1 [42]. Phylogenetic trees were constructed using maximum likelihood [43] and Bayesian methods [44]. The best-fit evolutionary model was determined using the Find Best-Fit Substitution Model (ML) in MEGA 5.1. For the Bayesian analyses the Tamura 3-parameter substitution model with gamma-distributed rate variation across all sites (T92+G) was used [45]. Four Markov chains were run and the chains were sampled every 100^th^ generation. To ensure convergence, replicate generations were run until the standard deviation of split frequencies fell below 0.05. The first 25% of the trees generated was discarded as burnin and trees that had not converged (split frequencies >0.05) were removed. Results of the analyses were summarized using a majority rule consensus tree, where support values represent nodal posterior probabilities.

#### (b) Ancestral character state reconstructions

We performed ancestral character state reconstructions in Mesquite 2.75 [46] under maximum likelihood, using the symmetrical Mk1 (one-parameter Markov k-state) model [47]. The *Panagrolaimus* phylogeny used for this analysis was a Bayesian 50% majority rule consensus tree (S3 Fig.). We further tested our results in Mesquite under maximum parsimony. The results were consistent with those obtained using the MK1 model and are not presented. Anhydrobiotic survival data were converted to binary characters as follows: survival following preconditioning for 72 h at 98% RH > 50% = 1; survival following preconditioning for 72 h at 98% RH < 50% = 0. These criteria were selected because the anhydrobiotic phenotypes of majority of strains had stabilized following 72 h preconditioning (S4 Fig.) and the phylogenetic signal at this time point was strong (Table 2). The three isolates coded 0 were *P. paetzoldi* (0% survival); *P*. sp. JU765 (0% survival) and *P*. sp. JU765 (20.1% survival); these three isolates are the sole members of PCA group 1. The freezing survival data for both cold acclimated and non acclimated nematodes were converted to binary characters as follows: percentage survival < 20% = 0; percentage survival > 20% = 1.

**Table 2:** Estimation of phylogenetic signal in *Panagrolaimius* freezing-tolerance and anhydrobiosis phenotypes, based on fit to a Brownian motion model of trait evolution estimated using Pagel’s *λ* metric [43] and the caper package [48].

Trait	*λ*	95% Confidence Interval	Likelihood Ratio Tests	
			*λ* = 0	*λ* = 1
*Freezing Tolerance*				
Not acclimated	0.80	(0.47–0.94)	<0.001	<0.001
Cold acclimated (10°C, 10d)	0.88	(0.47–0.97)	<0.01	<0.001
*Anhydrobiosis*				
Preconditioning (98% RH, 0h)	0.63	(0.16–0.92)	<0.01	<0.001
Preconditioning (98% RH, 24h)	0.78	(0.44–0.93)	<0.001	<0.001
Preconditioning (98% RH, 48h)	0.82	(0.51–0.95)	<0.001	<0.001
Preconditioning (98% RH, 72h)	0.82	(0.51–0.95)	<0.001	<0.001
Preconditioning (98% RH, 96h)	0.99	(0.97–1.0)	<0.001	N.S.

#### (c) Phylogenetic signal

**Fig 1:**
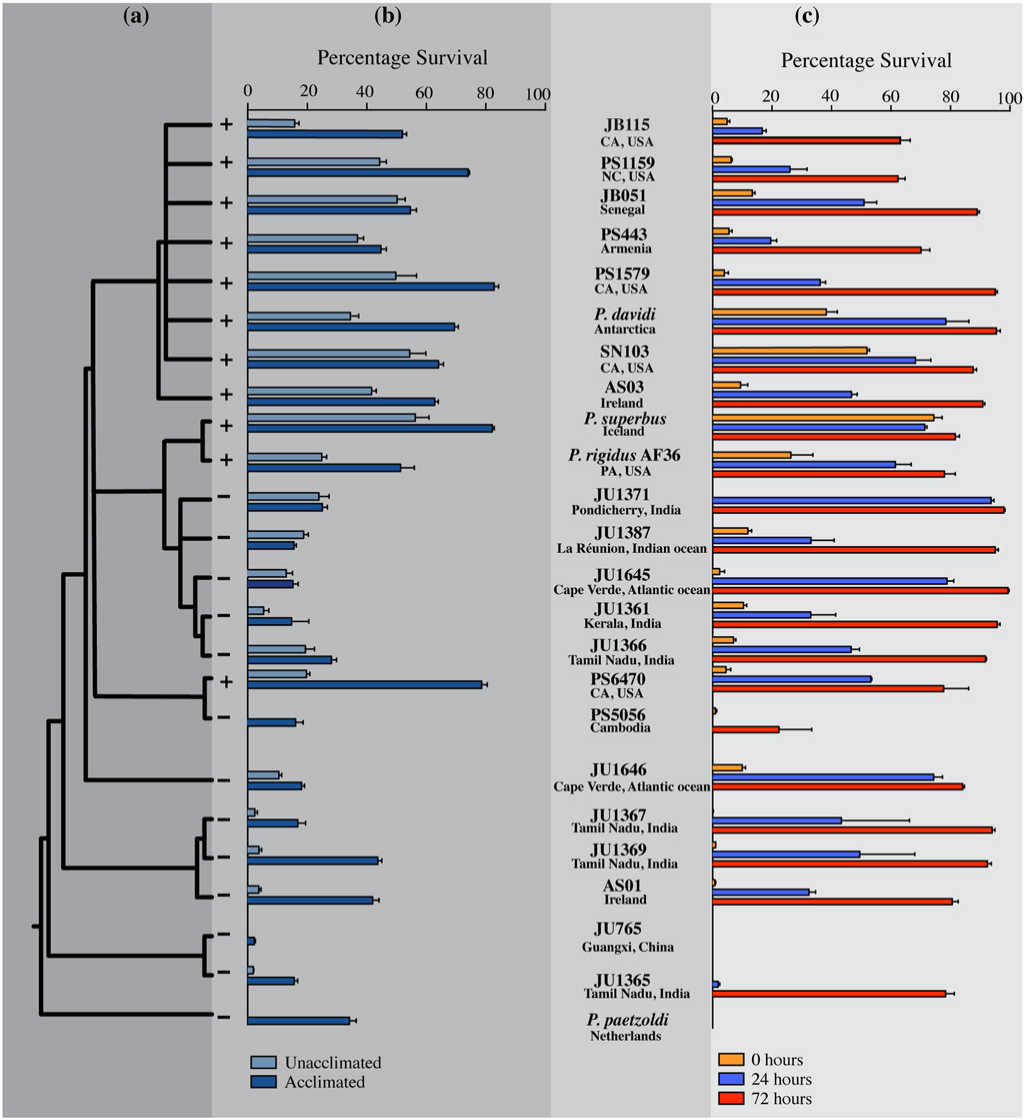
The phylogenetic relationships, anhydrobiotic and freezing tolerance phenotypes of 24 *Panagrolaimus* taxa. (a). Bayesian 50% majority rule consensus tree derived from sequences from the rDNA D3 expansion region *(see S3 Fig. for branch lengths for the phylogeny)*. (b). Freezing tolerance phenotypes. Unacclimated nematodes were cultured at 20°C, acclimated nematodes were cultured at 10°C for 10 d prior to freezing. Data are the means ± SEM of four biological replicates. Strains whose unacclimated tissue extracts inhibit ice crystal growth are indicated by +; those whose tissue extracts did not inhibit ice crystal growth are indicated by-. (c). The effect of preconditioning at 98% RH for 0, 24 or 72h on anhydrobiotic survival (see S4 Fig. for additional preconditioning time-points). Data are the means ± SEM of three biological replicates.

The extent to which the anhydrobiosis and cryotolerance phenotypes of the panagrolaimid taxa accorded with the topology and branch lengths of the rDNA D3 phylogeny (Fig. 1; S3 Fig.) was investigated using Pagel’s ***λ*** phylogenetic signal statistic [48]. Under a Brownian motion (BM) model of evolution, trait variance among species increases in direct proportion to their time of independent evolution, which is measured by the length of the branches separating them in a phylogeny [49]. Thus the phenotypes of members of lineages that have recently diverged tend to be more similar to each other than to members of more distantly related lineages. For a given phylogenetic tree and associated trait data the ***λ*** phylogenetic signal statistic is estimated from a phylogenetic variance co-variance matrix (i.e. the expected trait value in a given species is proportional to the total length of the tree, while the trait co-variance for each pair of species in the phylogeny is proportional to their shared evolutionary history which is given by the sum of their shared branch lengths) [50]. A maximum likelihood approach is then used to find the value of ***λ*** that best explains the variation for a continuous trait among the taxa at the tips of a phylogeny [48]. Pagel’s ***λ*** varies continuously from 0 to 1 with a ***λ*** value of 1 indicative of strong phylogenetic signal, i.e. the specified tree and a BM model of evolution fit the data well, while ***λ*** = 0 indicates a lack of phylogenetic signal, i.e. the traits under consideration are best represented by a “star” phylogeny where all species are equally distantly related to each other. Several evolutionary processes approximate a BM model [51,52]. These include genetic drift (neutral evolution), directional selection, fluctuating directional selection (where the optimal value is changed) and punctuated change. Scenarios which give rise to low phylogenetic signal are when a trait varies randomly across a phylogeny, or when distantly related species converge on a similar trait value (convergent evolution), or closely related species exhibit notably different trait values (adaptive radiation) or when the phenotype is influenced by the environment. To mitigate possible environmental effects, all the panagrolaimaid strains in our study were cultured and assayed under standard conditions. We estimated ***λ*** using the plgs function in the caper package [53]. The caper package calculates confidence intervals of the ML estimate and it incorporates log likelihood ratio tests of the significance of the estimated value of ***λ*** under the hypothesis ***λ*** = 0 and under the hypothesis ***λ*** = 1, with df = 1.

#### (d) Correlation analyses

The relationship between the survival of the cold acclimated nematodes and those that survived desiccation (preconditioning: 98% RH, 96 h) was investigated using Spearman’s non-parametric correlation test using the *GraphPad* Prism statistical package. To consider the impact of phylogeny on trait correlations within the PCA groups 3 and 4 from temperate and polar regions we generated an rDNA D3 phylogeny for these PCA Groups using a ClustalW2 multiple sequence alignment [41] and Bayesian phylogeny reconstruction methods [43], as described above. We then used a Phylogenetic Generalised Least Squares (PGLS) approach which simultaneously estimates Pagel’s *λ* along with the regression parameters to provide the necessary correction for trait covariance based on the phylogenetic signal of the data [50]. This analysis was carried out using the plgs function in the caper package [53] using the default scaling parameters as estimated by the program.

## Results and Discussion

### Anhydrobiotic and Freezing-Tolerance Phenotypes Occur Widely in *Panagrolaimus* Species and Strains

We previously reported on the anhydrobiotic capacity of 11 strains of *Panagrolaimus* [14,54]. Here we present survival data for 24 *Panagrolaimus* strains, 18 of which were not included in previous studies. Among these are newly isolated strains from Ireland, southern India, La Réunion, Cape Verde and California (See S1 Table for strain provenances). Some isolates are from relatively exposed sites—moss on a building roof (*Panagrolaimus* sp. AS03); a roof rain-gutter (sp. AS01); the surface of a rotting tree stump (sp. SN103); others were recovered from soil, or leaf litter or from rotting fruit at the soil surface, while *Panagrolaimus* sp. PS443 from Armenia was isolated from dry soil after 8 years storage in the laboratory [55]. The phylogenetic relationships of these strains are presented in Fig. 1(a) and S3 Fig. The effect of preconditioning the nematodes at 98% RH for 0, 24 or 72h on their subsequent anhydrobiotic survival, when exposed to freshly activated silica gel for 48 h, is presented in Fig. 1(c). Data for additional preconditioning time-points at 98% RH are shown in S4 Fig. These data show that a capacity to tolerate extreme desiccation over activated silica gel is widespread among *Panagrolaimus* strains and species.

When 24 unacclimated strains of *Panagrolaimus* were frozen at a cooling rate of 3.02°C min^-1^ and stored at -80°C for 24 h prior to thawing, their freezing-tolerance phenotypes appear to form a continuum [Fig. 1(b)], with survival values ranging from 0% to 56%. Eight strains are excellent survivors of freezing, with 39% or higher of the unacclimated nematodes surviving. This group includes *P. davidi* from Antarctica whose freezing capacity has previously been documented [29,30,56], as well as *P. superbus* from Iceland, but it also includes isolates from temperate regions. These results show that freezing tolerance phenotypes comparable, or superior, to that of *P. davidi* are common among panagrolamids. Surprisingly several of the strains from the tropics and subtropics were also able to survive freezing, having unacclimated survival values ranging from 2.3% (*Panagrolaimus* sp. JU1365, Tamil Nadu, India) to 23.8% (sp. JU1371, Pondicherry, India). Cold acclimation at 10°C for 10 days improves the freezing tolerance of the majority of the strains tested, most notably those with poor freezing tolerance when unacclimated [Fig. 1(b)].

### Principal Component Analysis of Combined Anhydrobiotic and Freezing-Tolerance Datasets Reveals Four Phenotypic Groups

**Fig 2:**
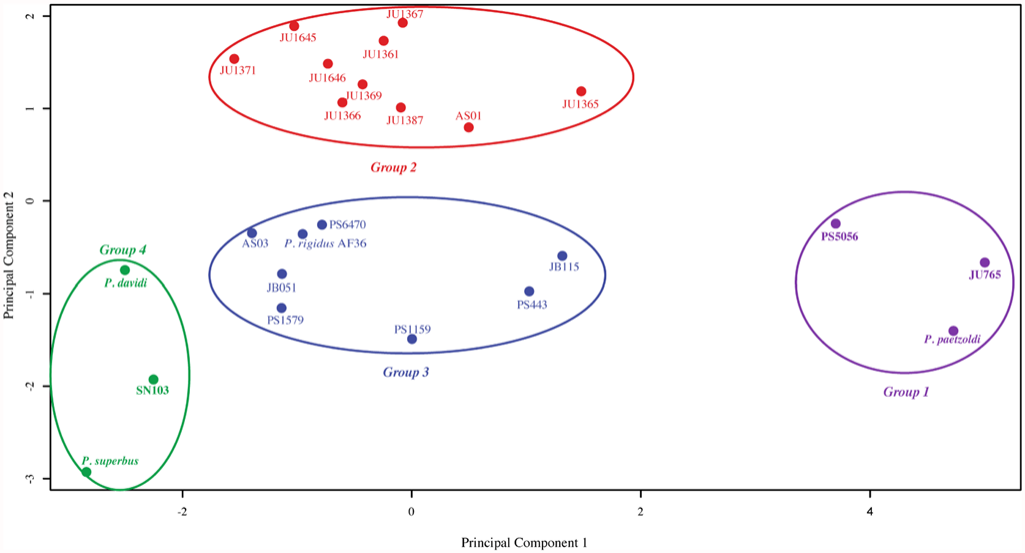
Principal component analysis of the desiccation and freezing survival data for 24 *Panagrolaimus* strains. The distribution of the strains in the plane formed by Principal Component 1 and Principal Component 2 is shown. These components, which correspond respectively to 57.34% and 24.86% of the variance, discriminated between four phenotypic groupings.

Principal component analysis (PCA) showed that two components explained 82.2% of the variability of the combined desiccation and freezing-tolerance phenotypes (S2 Table). These two components discriminated between four Groups (Fig. 2; S3 Fig.). The three strains in PCA Group 1 are desiccation sensitive and are freezing sensitive when unacclimated. Group 2 comprises *Panagrolaimus* sp. AS01 from Ireland, along with nine *Panagrolaimus* isolates with JU designations (from regions as different and distant as, for example, Southern India and Cape Verde in the Atlantic ocean). These nematodes have excellent “slow-dehydration” phenotypes, with maximal desiccation tolerance being achieved following preconditioning at 98% RH for 72–96 h. The survival values of 96 h preconditioned Group 2 nematodes range from 74.6% for JU1365 (from Tamil Nadu), to 95.7% for JU1361 (Kerala), but their freezing tolerance is moderate [Fig. 1(b)], ranging from 2.4% (JU1367, Tamil Nadu) to 24.9% (JU1371, Pondicherry) for non-acclimated nematodes and from 14.5% (JU1361) to 43.7% (JU1369, Tamil Nadu) for cold acclimated nematodes. PCA Group 3 contains slow-dehydration strategist nematodes with robust desiccation and freezing tolerances. Their maximal preconditioned desiccation survival values range from 67.1% (PS443 Byurakan, Armenia) to 92.6% (PS6470, Mid Mojave Desert, Ca, USA) and their cold-acclimated freezing survival values range from 44.7% (PS443) to 82.6% (PS1579, Botanic Gardens. San Mario, Ca). Group 4 strains (*P. superbus*, Surtsey, Iceland; *P. davidi*, Antarctica and *Panagrolaimus* sp. SN103 from a tree stump in the Cascade Mountains, Ca, USA) can withstand immediate exposure to extreme desiccation over activated silica gel and also have excellent freezing tolerance, with freezing survival values ranging from 64.1% survival (SN103) to 82.1% (*P. superbus*) for cold-acclimated nematodes.

### Freezing Tolerance in *Panagrolaimus* is Associated with a Capacity to Inhibit Ice Growth and Recrystallization, but not with Thermal Hysteresis.

#### Tissue extracts from several Panagrolaimus spp. can inhibit the growth of ice crystals

**Fig 3:**
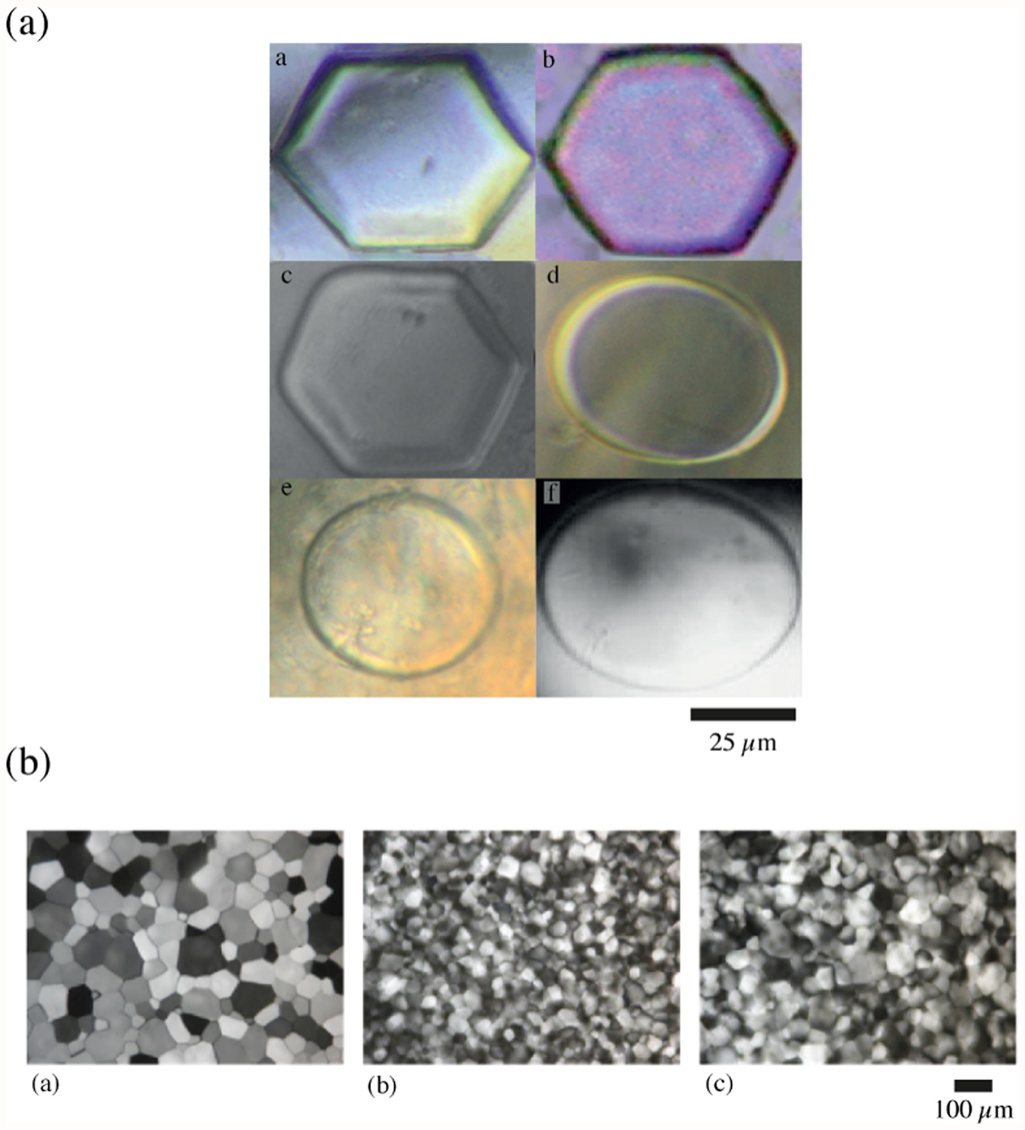
The effect of tissue extracts from unacclimated *Panagrolaimus* strains on the growth, morphology and recrystallization of ice crystals. Panel (a). Crystals formed in the presence of extracts from freezing-tolerant strains (a-c) have a distinct faceted morphology characteristic of the activity of ice-active proteins; ice crystals formed in the presence of extracts from a freezing sensitive strains (d, e) and in buffer controls (f) are cylindrical, indicating an uninhibited growth of the crystals. a: *P. davidi*; b: *P. superbus*; c: *Panagrolaimus* sp. PS1159; d: *Panagrolaimus* sp. JU765; e: *Panagrolaimus* sp. JU1367; f: S buffer (0.1 M NaCl, 0.05 M potassium phosphate, pH 6.0). Scale bar = 25 μM). Panel (b). Splat frozen samples of a: Tris buffer (25 mM Tris HCl, pH 8.6) and tissue extracts from b: *Panagrolaimus* sp. PS1159 and c: *Panagrolaimus* sp. SN103 following annealing at -8°C for 30 min. Scale bar = 100μM.

Extracts from *P. davidi* contain an ice-active protein (IAP) which inhibits the growth and recrystallization ice crystals [30]. Using freezing-point nanolitre osmometry, we tested the ability of tissue extracts from 24 unacclimated *Panagrolaimus* strains, including *P. davidi*, to inhibit the growth of a single seed ice crystal [Fig. 3(a); S5 Fig.]. In water or buffer, ice normally grows on the prism planes (along the horizontal *a* axes) of the crystal lattice, with little perpendicular growth on the basal plane (along the *c* axis), so that the ice crystals have the appearance of flat, round or irregular discs [27]. The majority of IAPs preferentially bind to the prism plane (S6 Fig.), inhibiting the growth of ice along the *a* axes and creating hexagonal discs [27]. When assayed at a protein concentration of 2 μg μL^-1^ the ice crystals formed from extracts of *P. davidi* and ten other *Panagrolaimus* strains were hexagonal in shape and inhibition of ice growth on the *a* axes of these crystals was evident from their truncated pyramidal growth patterns. Ice crystals formed from extracts from the remaining strains had the appearance of flat, round discs, indicative of unrestricted ice growth along the *a* axes. Fig. 1 (b) shows that the strains whose unacclimated tissue extracts inhibit ice-crystal growth have robust freezing tolerance (>20% survival following exposure to -80°C for 24 h) and that extracts from all members of the clade that contains *P. davidi* are able to inhibit the growth of ice crystals.

Ice recrystallization (the annealing of small crystals with larger ones) is thermodynamically favoured [57] and the large ice crystals which result cause mechanical damage to frozen cells and tissues. Recrystallization inhibition assays for extracts from two freezing-tolerant strains from the *P. davidi* clade (*Panagrolaimus* sp. SN103 and sp. PS1159) show that, similar to *P. davidi*, these extracts also possess recrystallization inhibition activity [Fig. 3(b)], consistent with our nanolitre osmometer results on the inhibition of ice crystal growth. The mean crystal diameters were as follows: buffer controls, 125.6±2.3 μM; *Panagrolaimus* sp. 1159, 38.2±1.3 μM and *Panagrolaimus* sp. SN103, 97.1±3.8 μM (one way ANOVA: *F* = 285.8, *p*<0.001).

#### Freezing tolerant *Panagrolaimus* spp. lack thermal hysteresis activity

Some fish and freeze-avoiding insects rely on antifreeze IAPs to lower the freezing temperature of their supercooled body fluids to a point below the temperature of the ambient environment [19,20], an activity known as thermal hysteresis (TH). In contrast, the ice-active proteins that occur in freezing-tolerant plants [27] and arthropods [58,59], as well as in *P. davidi* [30], lack significant TH activity. In these freezing-tolerant organisms the IAPs function to inhibit the growth and recrystallization of ice crystals [27,59], but they do not affect the freezing point of the tissue fluids. When monitoring the growth and melting of ice crystals from *Panagrolaimus* tissue extracts using nanolitre osmometry, no evidence of a significant TH effect was apparent for extracts from any of the 24 strains tested. TH measurements for extracts from two freezing-tolerant strains from the *P. davidi* clade (*Panagrolaimus* sp. SN103 and sp. PS1159) prepared using three buffer systems are presented in Table 1. The TH activity measured for the buffer controls (0.02–0.03°C) was in the same range as that of the tissue extracts, as has previously been observed by Wharton *et al*. for *P. davidi* [30]. Thus, lacking TH activity to lower the freezing point of their tissue fluids, the ability of unacclimated freezing-tolerant *Panagrolaimus* to inhibit the growth and recrystallization of ice during freezing and thawing is likely to be an important adaptation in protecting their tissues against freezing-related tissue damage.

### Cryoprotective Dehydration Versus Intracellular Freezing in *Panagrolaimus*

When the ambient aqueous medium freezes *P. davidi* may undergo either cryoprotective dehydration or intracellular freezing. The outcome depends on the ice nucleation temperature and the cooling and freezing rates of the ambient medium [56]. If the external medium freezes at high subzero temperatures (-1 to -2°C) *P. davidi* undergoes cryoprotective dehydration, losing water from its tissues because the vapour pressure of the external ice is lower than that of supercooled water at the same temperature [16]. When the ambient medium freezes at lower temperatures (-5°C or lower) ice propagation through *P. davidi* is rapid, both extracellular and intracellular compartments becoming frozen [56]. Similarly Convey and Worland [60] found that seven species of Antarctic nematodes were sensitive to exogenous ice nucleation, the nematodes freezing at high subzero temperatures (-2 to -5°C), which were above the supercooling points of their body fluids. In our study it also seems likely that ice nucleation of the ambient medium at -4.6°C (S1 Fig.) would cause the nematodes to undergo rapid inoculative freezing. The water loss that occurs during cryoprotective dehydration in nematodes and Collembola is associated with body shrinkage [18,56,61,62]. However photomicrographs of frozen *Panagrolaimus* taken immediately following thawing show no evidence of shrinkage in either the freezing-tolerant or freezing-sensitive strains (S7 Fig.), a strong indication that the tested *Panagrolaimus* strains did not undergo cryoprotective dehydration.

### Phylogenetic Signal

The phylogenetic signal for the anhydrobiosis and freezing-tolerance phenotypes was high (Table 2), an indication of congruence between these phenotypes and the topology and branch lengths of the rDNA D3 phylogeny (S3 Fig.). The signal for the anhydrobiosis phenotypes increased with the length of preconditioning time spent at 98% RH, ranging from *λ* = 0.63 for control nematodes to *λ* = 0.99 for nematodes which had been preconditioned at 98% RH for 96h. The large 95% confidence interval for non-preconditioned control nematodes most probably reflects the low survival values obtained for many panagrolaimid taxa in the absence of preconditioning at 98% RH. High phylogenetic signal was obtained for freezing-tolerance phenotypes in both cold-acclimated and non-acclimated nematodes, indicating that closely-related species exhibit similar trait values, a trend that is also evident from Fig. 1. Two large-scale studies of phylogenetic signal in a variety of vertebrate and invertebrate taxa have shown that the traits that exhibited the highest signals were body size and morphology followed by life history and physiology, with behaviour traits having the lowest phylogenetic signals [50,63]. Thus low phylogenetic signal is often equated with evolutionary lability and strong signal with evolutionary conservatism [63,64]. Among invertebrates, Kellermann *et al*. [65] recorded high phylogenetic signals for cold-tolerance phenotypes in *Drosophila* (Pagel’s *λ* = 0.76 for females and *λ* = 0.83 for males), and in NW European land snails *λ* values of 0.96–1.0 were obtained for traits associated with cold-hardiness, including the temperature of crystallization (spontaneous freezing) of the snails [66]. By contrast Kellermann *et al*. [65] found that the phylogenetic signal for desiccation resistance in *Drosophila* (scored as survival time (h) in dry air at 20°C) was low (*λ* = 0.17 for females and *λ* = 0.19 for males), which the authors interpret as suggesting that in *Drosophila* “desiccation resistance evolves rapidly and free from phylogenetic restrictions”. Although a high phylogenetic signal does not yield information about the evolutionary process (selection, stasis or drift) that shaped the anhydrobiosis and freezing tolerance phenotypes in *Panagrolaimus*, the general congruence between these phenotypes with the topology and branch lengths of the rDNA D3 phylogeny is indicative of the phylogenetic stability of these traits, particularly for anhydrobiotic survival in strains preconditioned at 98% RH for 72–96 h. Phylogenetic stability of “slow-dehydration” anhydrobiosis phenotypes in *Panagrolaimius* may have resulted from stabilizing selection of an adaptively optimum phenotype and/or from phylogenetic niche conservatism [67,68], where diverging lineages retain similar niche-related traits following dispersal to new habitats.

### Is There a Correlation between Freezing-Tolerance and Anhydrobiosis in *Panagrolaimus*?

**Fig 4:**
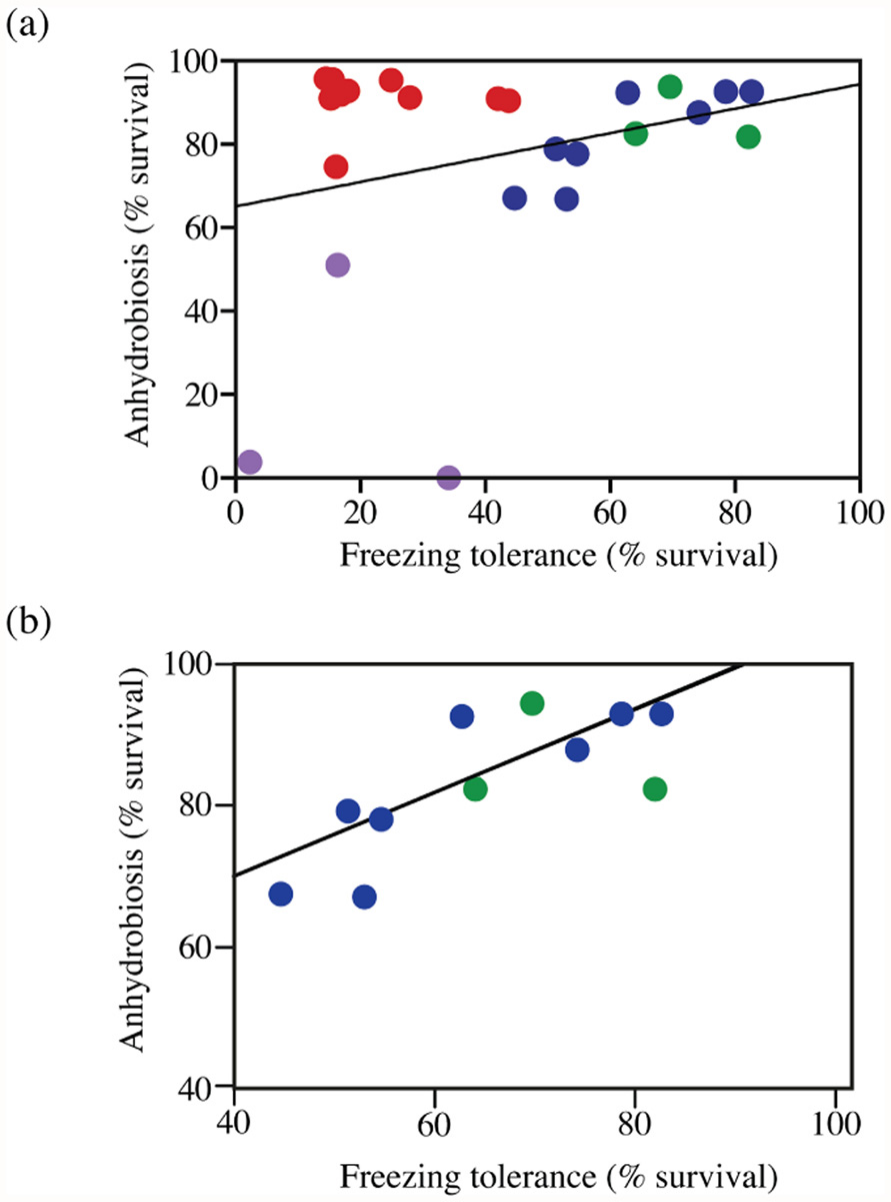
Correlation between freezing-tolerance and anhydrobiosis in *Panagrolaimus*. (a) Spearman rank correlation between freezing-tolerance and anhydrobiosis in all 24 *Panagrolaimus* strains (r = 0.0033, N.S.). (b) Spearman rank correlation between freezing-tolerance and anhydrobiosis in *Panagrolaimus* PCA Group 3 and Group 4 strains (Spearman’s *r* = 0.718, *p* < 0.05). The data points are shaded as follows: PCA Group 1, purple; Group 2, red; Group 3 blue; Group 4 green.

A correlation between freezing-tolerance and anhydrobiotic phenotypes might occur in *Panagrolaimus* because of an overlap in the biochemical protection responses of the nematodes to desiccation and freezing; or because of their shared phylogeny; or because they occupy an environment where correlated selection for freezing-tolerance and anhydrobiosis may occur. Studies in insects that overwinter in temperate and polar environments frequently show evidence of cross-tolerance between the physiological responses to cold stress and desiccation stress, where the mechanisms that protect against one of these stresses also provide protection against the other (reviewed by Sinclair et al. [69]). Correlation analysis [Fig. 4(a)] shows that the freezing and anhydrobiotic phenotypes of all 24 *Panagrolaimus* strains are not correlated (Spearman’s *r* = 0.0033, *p* = 0.99), indicating that the biochemical and physiological responses of these nematodes to desiccation and freezing are unlikely to provide cross-tolerance. It is noteworthy that the nematodes in PCA Group 2 that possess the highest desiccation tolerance have substantially weaker freezing tolerance than PCA Group 3 and 4 nematodes [Fig. 4(a)]. PCA Group 2 nematodes are tropical and sub-tropical in origin, whereas the isolates from PCA Groups 3 and 4 were isolated in temperate and polar regions (with the exception of JB051 from Senegal). It is unlikely that the tropical and sub-tropical *Panagrolaimus* isolates have been exposed to correlated selection for freezing-tolerance and anhydrobiosis in their recent evolutionary history, however the isolates from temperate and polar regions are more likely to experience such correlated selection. PCA Groups 3 and 4 strains show a strong positive relationship between their freezing-tolerance and anhydrobiotic phenotypes [Fig. 4(b)], (Spearman’s *r* = 0.718, *p* = 0.016*). To assess whether this correlation may be the result of correlated selection for these traits, we generated an rDNA D3 phylogeny for PCA Groups 3 and 4 and we used a PGLS approach which estimates Pagel’s λ simultaneously with the regression parameters to provide a correction of trait covariance based on the phylogenetic signal of the data [50]. The results obtained (PGLS Adjusted *R* ^*2*^ = 0.5568, *p* = 0.005**, λ = 0) also show that freezing-tolerance and anhydrobiosis are correlated traits in the *Panagrolaimus* strains from PCA Groups 3 and 4. However these results need to be interpreted conservatively because, given the estimated λ = 0 value for these traits in the PCA Group 3 and 4 phylogeny, the PGLS correlation was estimated under the assumption that all the panagrolaimid isolates in PCA Groups 3 and 4 are equally distantly related to each other.

### Ancestral Character State Reconstruction of the Freezing-Survival and Anhydrobiotic Phenotypes during *Panagrolaimus* Evolution

**Fig 5:**
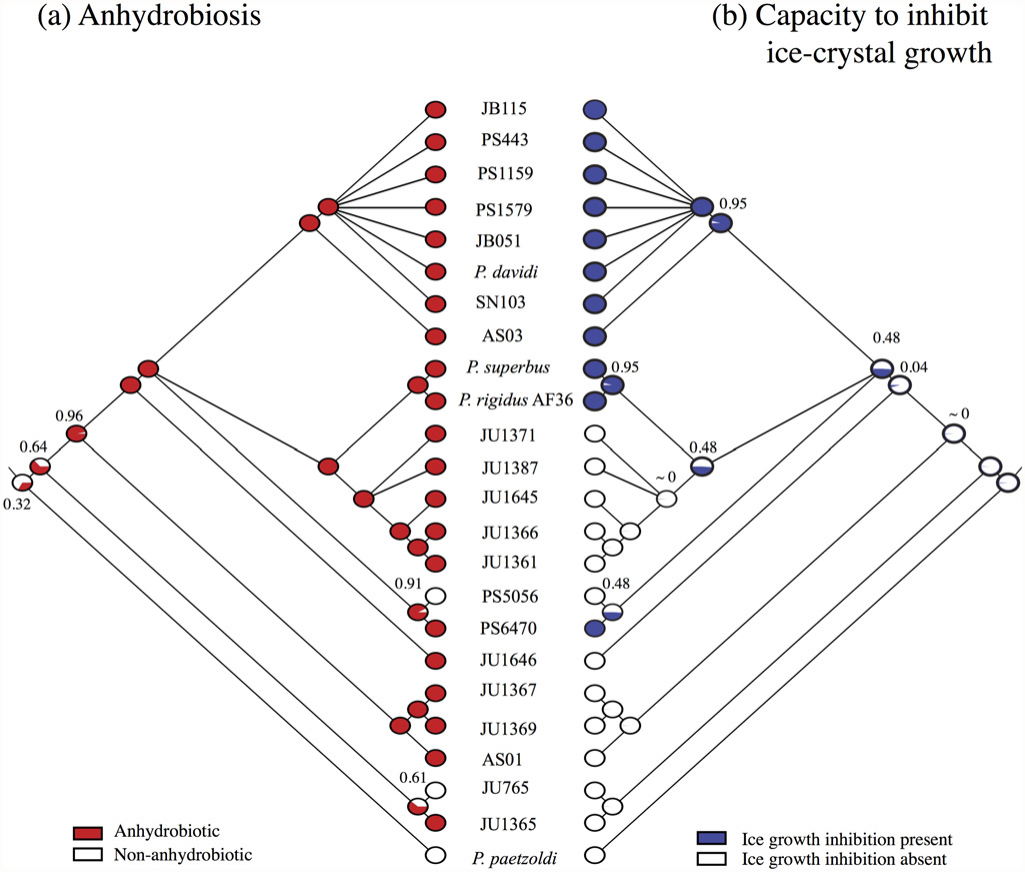
Maximum likelihood ancestral state reconstructions of anhydrobiotic (a) and ice-growth inhibition phenotypes (b) in *Panagrolaimus* under the assumption that all transition rates are equal and branch lengths are proportional to time. At each node a pie chart represents the proportional (0–1) likelihood of each ancestral character state, coloured as in the key. Anhydrobiotic survival data were converted to binary characters as follows: survival following preconditioning at 72 h > 50% = 1; survival following preconditioning at 72 h < 50% = 0.

Nematodes are essentially aquatic animals that need to be fully hydrated and covered in a water film for normal activity. Thus a large majority of nematode species are highly sensitive to desiccation and osmotic stress [10]. Ancestral character state reconstructions [Fig. 5(a)] show that an anhydrobiotic phenotype evolved deep in the phylogeny of *Panagrolaimus*. The probability that the ancestor of the clade containing *Panagrolaimus* spp. AS01, JU1367 and JU1369 was anhydrobiotic is significant (*p* = 0.96). A lower, but still relatively high (*p* = 0.64), probability exists that the more basal ancestor of the clade containing also *Panagrolaimus* spp. JU765 and JU1365 was also anhydrobiotic. Thus the lack of anhydrobiosis in *Panagrolaimus* sp. PS5056 most likely represents a secondary loss. The anhydrobiotic strains located in the early-diverging *Panagrolaimius* lineages have potent slow-dehydration phenotypes (PCA Group 2), indicating that the ancestral nematodes required gradual environmental desiccation to induce the molecular changes necessary for successful entry into anhydrobiosis. From these early anhydrobiotes a capacity to respond to more rapid desiccation emerged.

The evolution of robust freezing tolerance (survival > 20%), along with a capacity to inhibit the growth and recrystallization of ice crystals, arose relatively recently in *Panagrolaimus* phylogeny [Fig. 1; Fig. 5(b)]. In the most unparsimonious reconstruction, a capacity to inhibit ice-crystal growth emerged three times independently: in the *davidi* group, in the *superbus* group and in *Panagrolaimus* sp. PS6470. However, an intriguing possibility is that *Panagrolaimus* sp. PS5056 might have lost the ability to inhibit the growth of ice crystals secondarily, as this taxon has also secondarily lost anhydrobiosis [Fig. 5(a)] and its sister taxon is both anhydrobiotic and freezing tolerant. Under this scenario, a capacity to inhibit ice-crystal growth would have evolved only once in the common ancestor of the *davidi*, *superbus* and *Panagrolaimus* spp. PS6470/PS5056 clades. This reconstruction would also imply that the ice growth-inhibiting phenotype was secondarily lost in the five JU strains that form sister clade of the *superbus* group. These five JU strains were isolated in the tropics (southern India, La Réunion and Cape Verde, S1 Table), however they possess intermediate levels of freezing tolerance, which may represent a relict of a more potent freezing-tolerant ancestral state.

**Fig 6:**
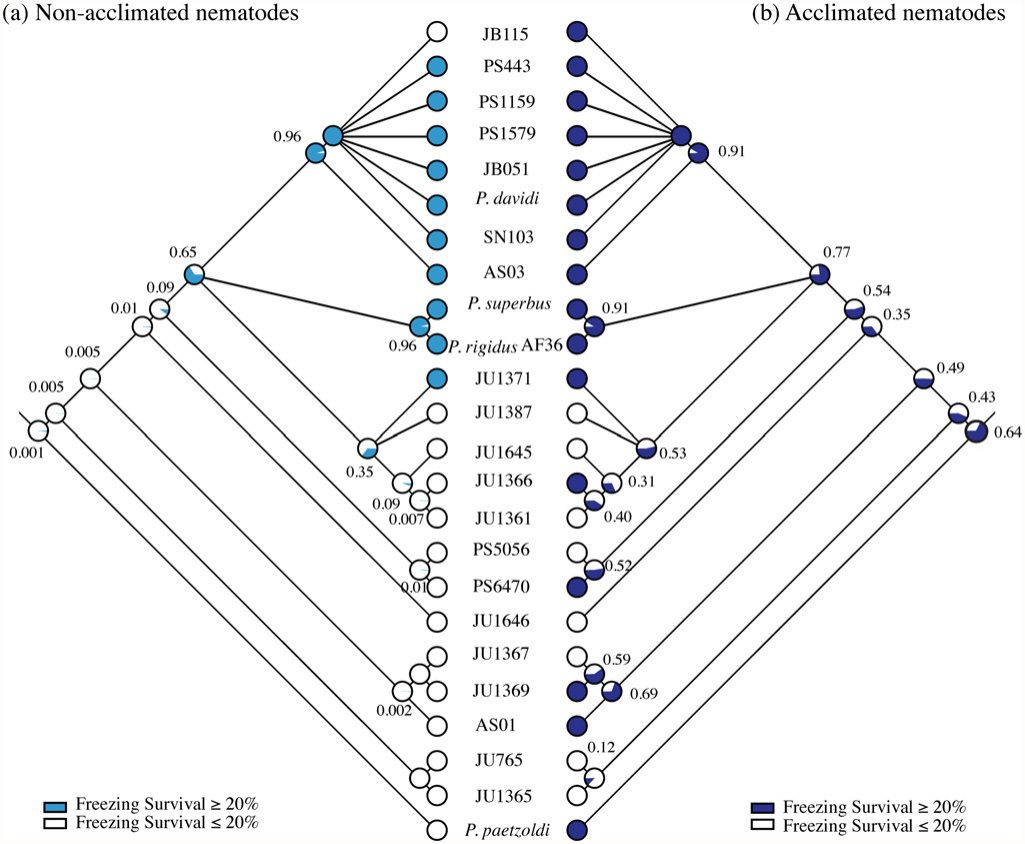
Maximum likelihood ancestral state reconstructions of freezing-tolerant phenotypes of (a) non-acclimated and (b) cold-acclimated *Panagrolaimus* species and strains, under the assumption that all transition rates are equal and branch lengths are proportional to time. At each node a pie chart represents the proportional (0–1) likelihood of each ancestral character state, coloured as in the key. Survival data were converted to binary characters as follows: freezing survival > 20% = 1; freezing survival < 20% = 0. The nematodes were cold acclimated on nematode growth medium (NGM) plates at 10°C for 10 days.

Freezing survival data [Fig. 1(b)] were converted to binary characters as follows: a) non-acclimated nematodes—survival < 20% = 0, n = 14, survival ranges 0–19.6%; survival > 20% = 1, n = 10, survival ranges 23.8–56.3%; b) cold-acclimated nematodes—survival < 20% = 0, n = 8, survival ranges 2.29–18.0%; survival > 20% = 1, n = 16 survival ranges 24.9–82.7%. We consider that the ten nematode isolates whose non-acclimated freezing survival exceeds 20% show evidence of robust freezing tolerance, with eight of these isolates having survival values in the range 35%- 56%. Included in this group are *P. davidi* from Antarctica (35% non-acclimated survival) and *P. superbus* from Iceland (56% non-acclimated survival). Ancestral character state reconstructions of the freezing-tolerant phenotypes of unacclimated *Panagrolaimus* [Fig. 6(a)] is congruent with the evolutionary history of the ice growth-inhibition phenotype postulated above. Cold acclimation improves the freezing tolerance of the majority of *Panagrolaimus* strains, and it has a marked effect on the survival of some strains from the early-stemming lineages that are freezing sensitive when unacclimated. Reconstructions of the ancestral character state of the freezing-tolerant phenotypes of cold-acclimated nematodes reveal a complex evolutionary pattern [Fig. 6(b)], suggesting that inducible proto-cryobiotic phenotypes may have evolved as independent lineage-specific events in several of the early-diverging *Panagrolaimus* species. In addition, this latter dataset provides further support (*p* = 0.77) for the hypothesis that the ancestor of the *davidi*, *superbus* and JU Cape Verde clades was freezing tolerant.

The branch lengths subtending the terminal taxa in the phylogenetic tree derived from sequences from the rDNA D3 expansion region (S3 Fig.) indicate that several of the early-stemming lineages are quite divergent from the *davidi*, *superbus* and Cape Verde clades. Gaining a better insight into the evolution of freezing tolerance and acclimation phenotypes in the early-diverging *Panagrolaimus* species will necessitate the isolation and characterization of additional *Panagrolaimus* isolates from the early-stemming lineages of the phylogeny, as well as the testing of outgroups from the 24 currently accepted genera within the family *Panagrolaimidae* [70]. Although our ancestral state reconstructions do not provide a clear insight into the evolution of freezing tolerance in the early-diverging *Panagrolaimus* species, our ancestral character state reconstructions clearly show that the evolution of freezing tolerance in the *davidi* and *superbus* lineages arose in an anhydrobiotic ancestor and that the common ancestor of the *davidi* clade possessed a robust freezing tolerance and an ability to inhibit the growth and recrystallization of ice crystals prior to the lineage-splitting and radiation of this clade and the dispersal of *P. davidi* to Antarctica.

### The Colonization of Antarctica by *P. davidi* and of Surtsey by *P. superbus*—Examples of “Ecological Fitting”?

Ancestral character state reconstructions show that the common ancestor of the *P. davidi* lineage and of the *P. superbus* lineage were freezing tolerant and also had the capacity to inhibit ice-crystal growth (Fig. 5). *P. superbus*, an early colonizer in a gull’s nest at Surtsey after the volcanic formation of this island, was most likely transported to the island as an anhydrobiotic propagule. The nematodes in the freezing-tolerant *P. davidi* clade are self-fertile, while the remaining nematodes in the phylogeny, with the exception of *Panagrolaimus* sp. JU765, are dioecious (S1 Table). A self-fertile mode of reproduction would facilitate the establishment of nematode populations following anhydrobiotic dispersal to new habitats. All members of the *P. davidi* clade have strong freezing-tolerance and anhydrobiosis phenotypes, along with the capacity to inhibit the growth of ice crystals. This combination of reproductive, anhydrobiotic and freezing-tolerant phenotypes is likely to have contributed to the wide geographic dispersal of the members of this clade and facilitated colonization of Antarctica by *P. davidi*. The Antarctic-specialist nematode *Scottnema lindsayae*, the sole member of a monotypic genus, is abundant and widespread where suitable ice-free soil occurs. In the laboratory its optimal growth temperature is 10°C [71], its reproductive cycle takes 218 days and its reproductive capacity declines if grown at higher temperatures [72]. By contrast, the optimal growth temperatures for *P. davidi* and *P. superbus* is ~25°C [73,74], and their life cycles are ~8 days at optimal temperature. These physiological and life-history traits do not show evidence of an evolved response to the polar environment and are more consistent with those of nematodes from a more seasonal temperate climate. The term “ecological fitting” was coined by Janzen [75] to describe the range expansion that may occur when a population acquires a robust genotype. In ecological fitting an organism interacts with its environment in a way that appears to indicate a shared evolutionary history, when the traits relevant to the interaction actually evolved elsewhere in response to different environmental conditions [76]. Our ancestral character state reconstructions suggest that the colonization of Antarctica by *P. davidi* and of Surtsey by *P. superbus* may be examples of the “ecological fitting” of pre-evolved freezing-tolerant phenotypes, together with a pre-evolved capacity to inhibit ice growth, to the prevailing abiotic conditions in these locations.

## Conclusions

*Panagrolaimus* nematodes evolved an anhydrobiotic phenotype at an early stage in their evolutionary history. The anhydrobiotic early-diverging lineages were slow-dehydration strategists, requiring gradual environmental desiccation for entry into anhydrobiosis. Robust freezing-tolerant phenotypes, along with the capacity to inhibit ice crystal growth arose more recently in *Panagrolaimus* phylogeny. Since all the early-stemming lineages and the majority of the tropical isolates have weak freezing tolerance phenotypes, the correlation between freezing tolerance and anhydrobiosis across the *Panagrolaimus* phylogeny is weak. The stronger correlation between freezing-tolerant and anhydrobiotic phenotypes observed in nematodes from temperate and polar regions is suggestive of correlated selection for these traits in these environments. Cold acclimation improves the freezing tolerance of the majority of *Panagrolaimus* strains and it has a marked effect on the survival of some of the early-stemming strains that are freezing sensitive when unacclimated. This phenotypic plasticity in a slow-dehydration anhydrobiote ancestor may have provided a suitable *milieu* for the evolution of more potent freezing tolerance in nematodes occupying freezing-prone environments. The acquisition of a mechanism to inhibit intracellular ice growth is likely to have been important step in the evolution of freezing tolerance in these proto-cryobiotic ancestors.

The common ancestors of the *davidi* and the *superbus* clades were anhydrobiotic and also possessed robust freezing tolerance, along with a capacity to inhibit the growth and recrystallization of ice crystals. *P. superbus* was an early colonizer at Surtsey after the volcanic formation of this island. Unlike other endemic Antarctic nematodes, the life history traits of *P. davidi* do not show evidence of an evolved response to polar conditions and are more consistent with those of nematodes from a more seasonal temperate climate. Thus we suggest that the colonization of Antarctica by *P. davidi* and of Surtsey by *P. superbus* is an example of a contingent effect resulting from the ecological fitting of two freezing-tolerant nematode species to polar abiotic conditions.

Several of the early-stemming lineages in the rDNA D3 phylogeny are quite divergent from the *davidi*, *superbus* and Cape Verde clades. Gaining a better insight into the evolution of freezing-tolerance and acclimation phenotypes in the early-diverging *Panagrolaimus* species will require the isolation and characterization of additional *Panagrolaimus* isolates from the early-diverging lineages of the phylogeny, as well as the testing of outgroups from the 24 currently accepted genera within the family *Panagrolaimidae*. Better resolution of the polytomy in the clade containing *P. davidi* can be achieved incorporating sequence data from additional genes, and further insight into the evolution of the *P. superbus* clade would benefit from the isolation and characterization of *Panagrolaimus* strains from Iceland and adjacent countries, such as Norway and Scotland. Overall, the data presented here show that panagrolaimid nematodes are an excellent model system to study the evolution of anhydrobiosis and freezing tolerance. They are also ideally suited for comparative high-throughput transcriptomic and genomic studies which would provide molecular insights into the molecular mechanisms involved in anhydrobiosis and freezing tolerance.

## Supporting Information

S1 Fig The cooling curve obtained when a one mL suspension of nematodes (2,000 nematodes mL^-1^ distilled water) was frozen in a -80°C freezer. (PDF)

S2 Fig The effect of acclimation temperature and length of acclimation on freezing survival in *Panagrolaimus superbus*. The nematodes were acclimated (a) on NGM agar plates containing a lawn of *E*. *coli* for different times and temperatures or (b) in water at different temperatures for 6 h prior to exposure in water to -80°C, at a cooling rate of 3.02°C min^-1^. After 24 h the nematodes were thawed and allowed to recover at 20°C for 24 h before their survival was determined. Survival values are the mean ± SEM of four biological replicates (* = *p*<0.05, ANOVA).
(PDF)

S3 Fig Hypothesis of phylogenetic relationships among strains and species *Panagrolaimus* derived from sequences from the rDNA D3 expansion region. Bayesian 50% majority rule consensus tree obtained using Tamura 3-parameter model of evolution, with gamma-distributed rate variation across all sites (T92+G) [45]. Branch supports (Bayesian posterior probabilities) are shown. The colour groupings correspond to PCA groups 1–4 (Fig. 2): Group 1, purple; Group 2, red; Group 3 blue; Group 4 green.
(PNG)

S4 Fig The effect of preconditioning at 98% RH on anhydrobiotic survival of 24 *Panagrolaimus* taxa. The nematodes were preconditioned at 98% RH for 0, 24, 48, 72 or 96 h, desiccated for 48 h over activated silica gel, rehydrated in distilled water for 24 h before survival was determined. Data are the means ± SEM of three biological replicates. The groupings distinguished by PCA of the combined freezing and anhydrobiosis phenotypes are indicated.
(PDF)

S5 Fig Effect of tissue extracts from unacclimated *Panagrolaimus* (grown on NGM agar plates at 20°C) on the growth and morphology of ice crystals of 19 strains of *Panagrolaimus*. The strains are A: AS03; B: *P. rigidus* AF36; C: SN103; D: JB115; E: PS1579; F: JB051; G: PS443; H: PS6470; I: *P. paetzoldi*; J: AS01; K: PS5056; L: JU1361; M: JU1365; N: JU1366; O: JU1369; P: JU1371; Q: JU1387; R: JU1645; S: JU1646; T: *Caenorhabditis elegans*. See S1 Table for the sources and geographic origins of the *Panagrolaimus* strains. Tissue extracts from unacclimated nematodes of PCA Groups 3 and 4 can inhibit ice crystal growth, while this capacity was not detected in any of the strains from PCA Groups 1 and 2. (scale bar 25 μm).
(DOCX)

S6 Fig A truncated bipyrimidal ice crystal (schematic). The majority of ice active proteins preferentially bind to the prism planes, inhibiting the growth of ice along these planes and creating hexagonal discs. Continued ice growth on the basal planes and continued binding to prism faces results in the formation of truncated bipyrimidal ice crystals. Figure adapted from Davies and Hew [25].
(PDF)

S7 Fig Photomicrographs of unfrozen controls and of frozen nematodes taken immediately post thawing. The nematodes are: *Panagrolaimus davidi* (Antarctica) and *P. superbus* (Surtsey, Iceland), both freezing tolerant species; *Panagrolaimus* sp. JU1646 (Cape Verde), a strain with weak freezing tolerance and *P. paetzoldi* (Netherlands), a freezing sensitive species. Scale bar: strains a, c and g, 500 μm; strains b, d, e, f and h, 200 μm.
(PDF)

S1 Table Source of the *Panagrolaimus* isolates used in this study. (DOCX)

S2 Table Principal component analysis of the anhydrobiotic and freezing-tolerance phenotypes of *Panagrolaimus* species and strains. (DOCX)

S3 Table GenBank accession numbers for the rDNA sequences used in *Panagrolaimus* phylogeny reconstructions. (DOCX)
