# Supplementary figures and images for: Anhydrobiosis and Freezing-Tolerance: Adaptations That Facilitate the Establishment of Panagrolaimus Nematodes in Polar Habitats
Source: PLoS One. 2015 Mar 6;10(3):e0116084. doi: 10.1371/journal.pone.0116084 (PMC4352009; doi:10.1371/journal.pone.0116084)

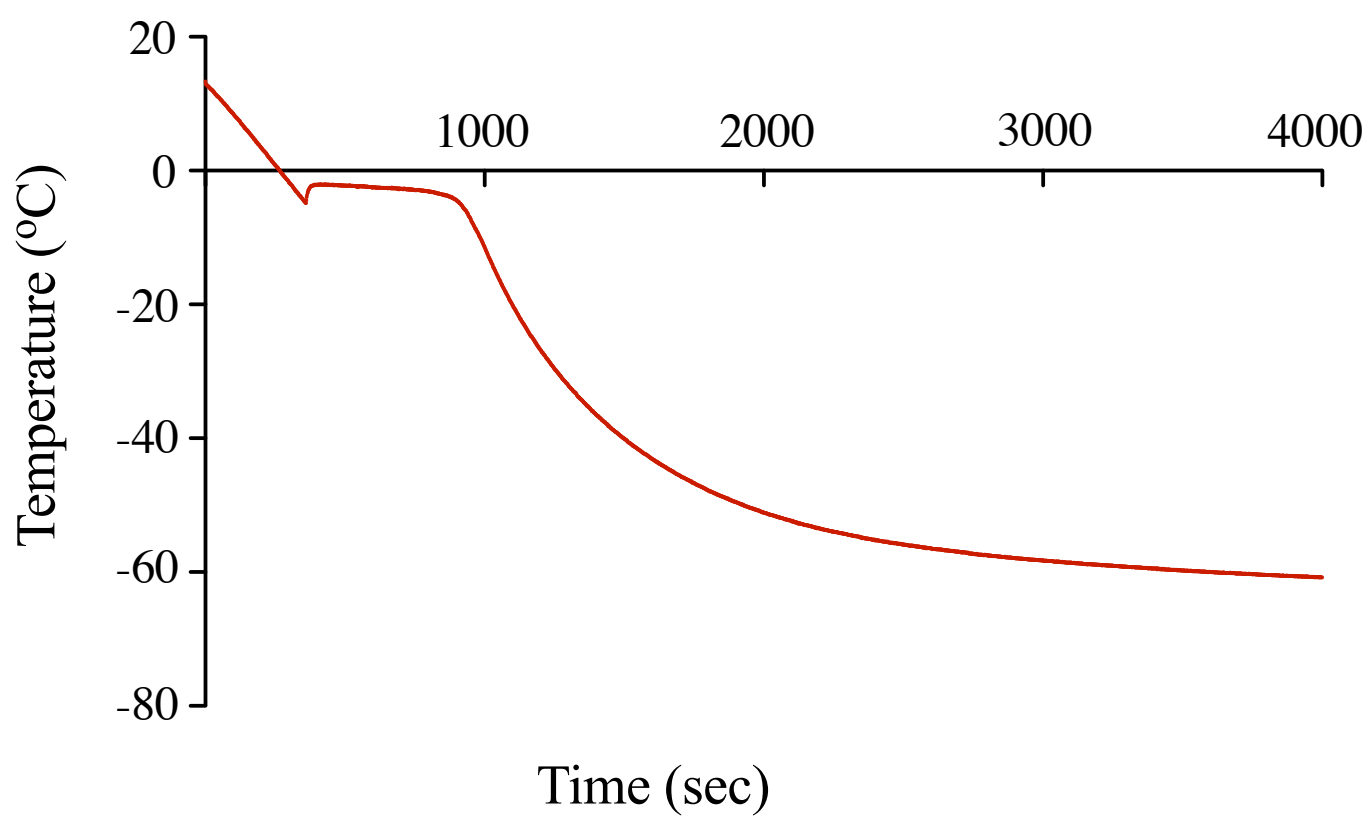

Supplement: S1 Fig — (PDF) [file pone.0116084.s001.pdf]

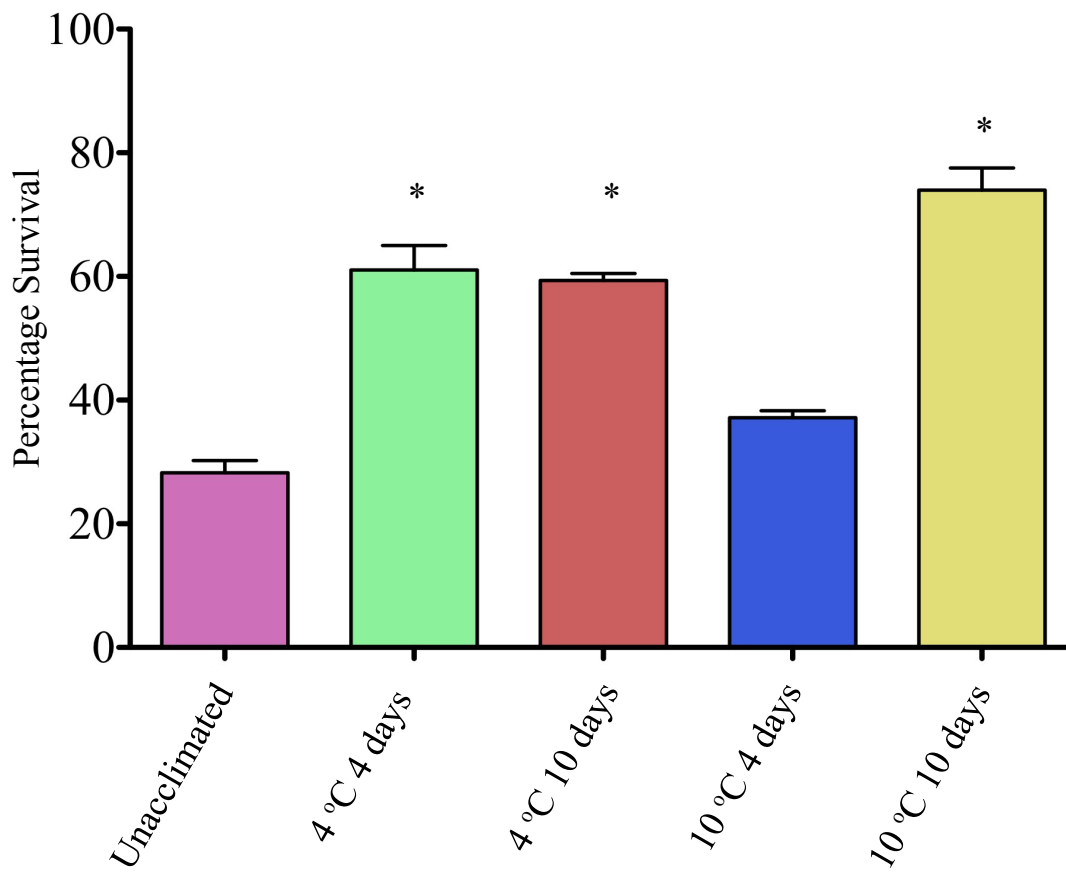

(a) Acclimation on NGM plates

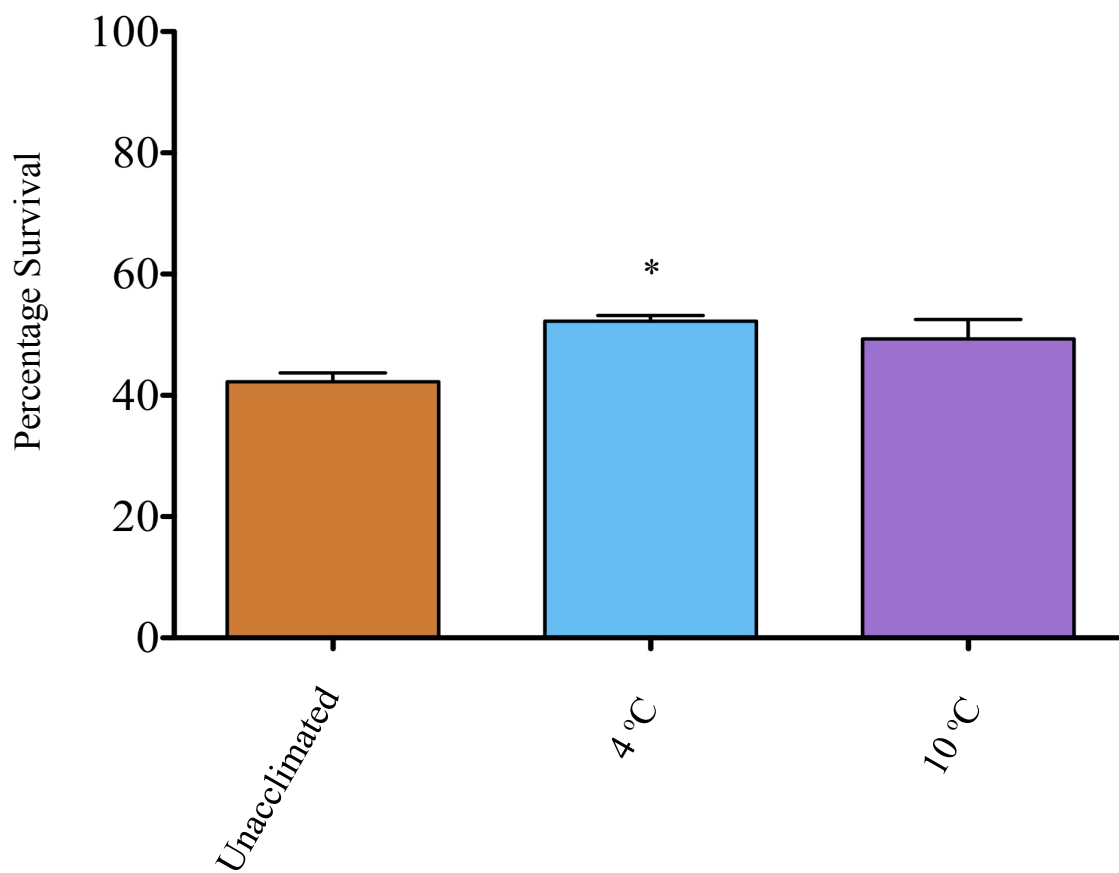

(b) Acclimation in water for 6 hours

Supplement: S2 Fig — The nematodes were acclimated (a) on NGM agar plates containing a lawn of E. coli for different times and temperatures or (b) in water at different temperatures for 6 h prior to exposure in water to -80°C, at a cooling rate of 3.02°C min-1. After 24 h the nematodes were thawed and allowed to recover at 20°C for 24 h before their survival was determined. Survival values are the mean ± SEM of four biological replicates (* = p<0.05, ANOVA). (PDF) [file pone.0116084.s002.pdf]

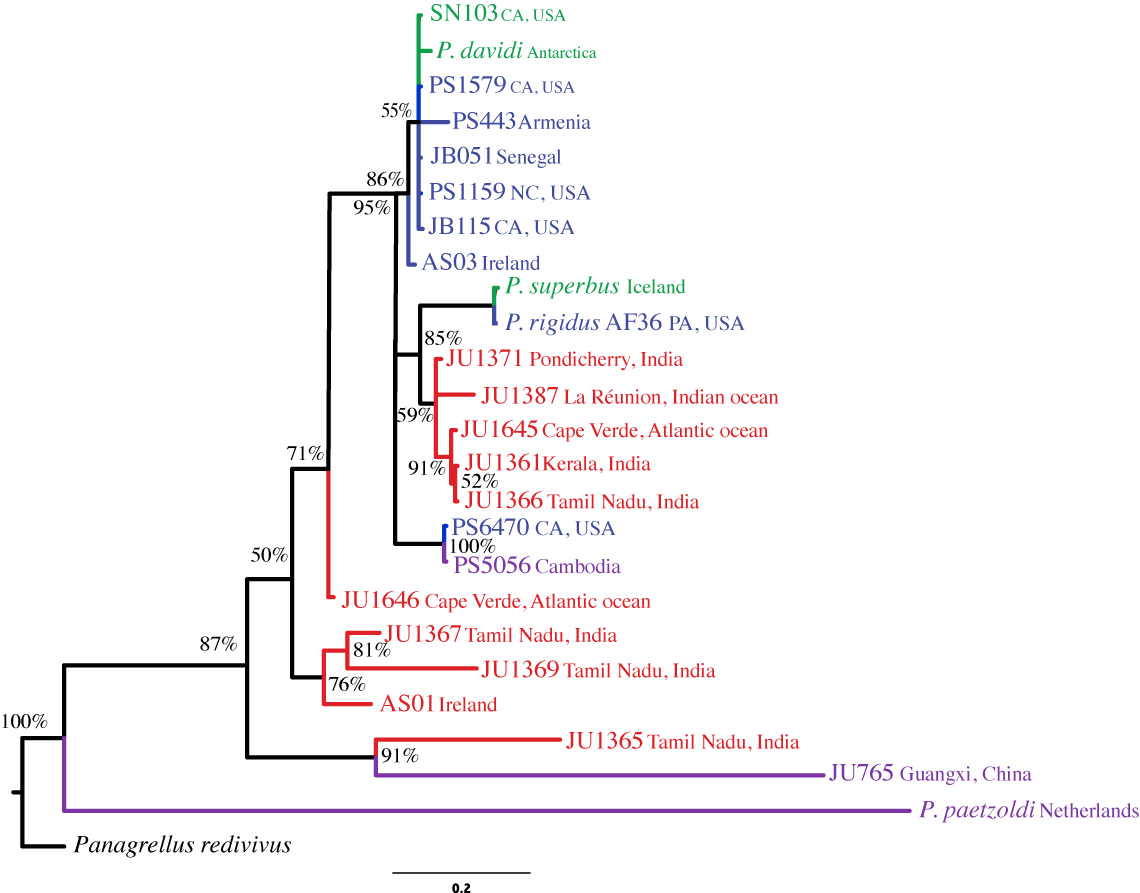

Supplement: S3 Fig — Bayesian 50% majority rule consensus tree obtained using Tamura 3-parameter model of evolution, with gamma-distributed rate variation across all sites (T92+G) [45]. Branch supports (Bayesian posterior probabilities) are shown. The colour groupings correspond to PCA groups 1–4 (Fig. 2): Group 1, purple; Group 2, red; Group 3 blue; Group 4 green. (PNG) [file pone.0116084.s003.png]

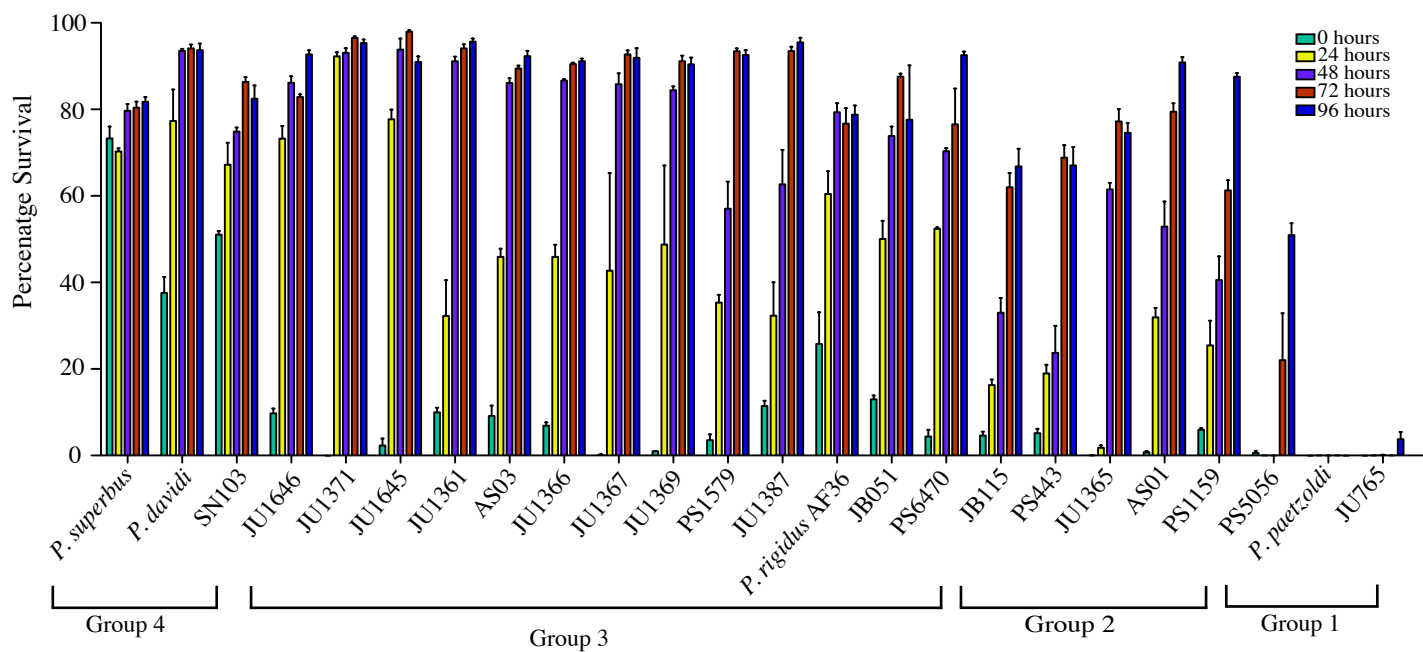

Supplement: S4 Fig — The nematodes were preconditioned at 98% RH for 0, 24, 48, 72 or 96 h, desiccated for 48 h over activated silica gel, rehydrated in distilled water for 24 h before survival was determined. Data are the means ± SEM of three biological replicates. The groupings distinguished by PCA of the combined freezing and anhydrobiosis phenotypes are indicated. (PDF) [file pone.0116084.s004.pdf]

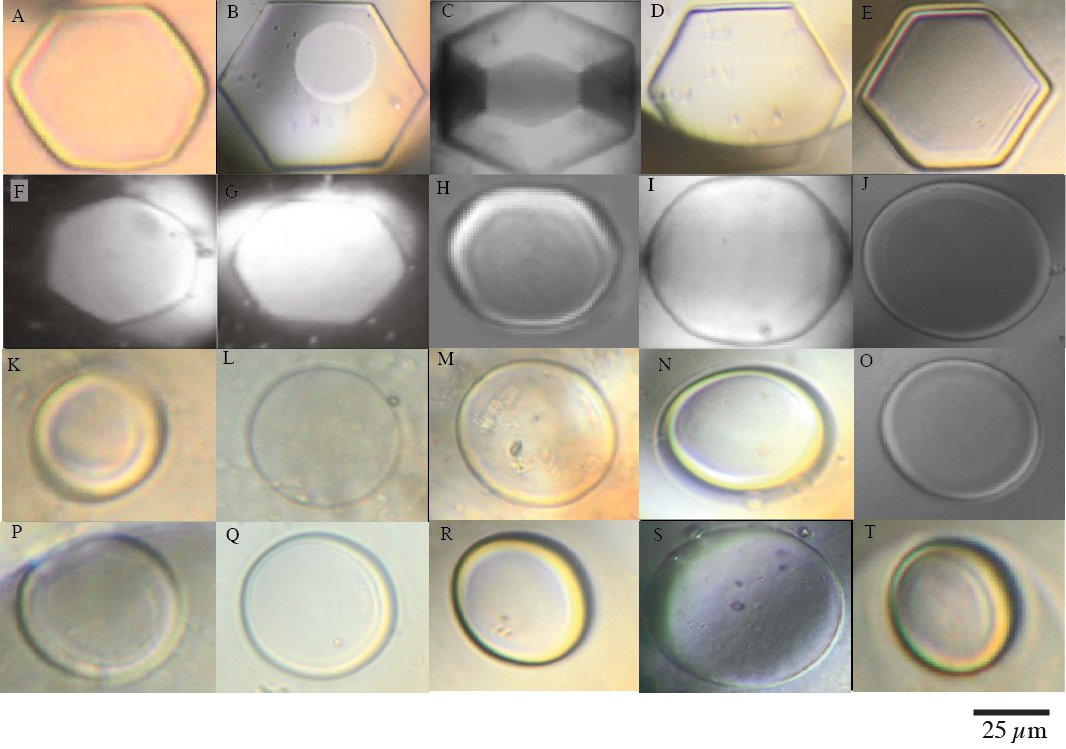

Supplement: S5 Fig — The strains are A: AS03; B: P. rigidus AF36; C: SN103; D: JB115; E: PS1579; F: JB051; G: PS443; H: PS6470; I: P. paetzoldi; J: AS01; K: PS5056; L: JU1361; M: JU1365; N: JU1366; O: JU1369; P: JU1371; Q: JU1387; R: JU1645; S: JU1646; T: Caenorhabditis elegans. See S1 Table for the sources and geographic origins of the Panagrolaimus strains. Tissue extracts from unacclimated nematodes of PCA Groups 3 and 4 can inhibit ice crystal growth, while this capacity was not detected in any of the strains from PCA Groups 1 and 2. (scale bar 25 μm). (DOCX) [file pone.0116084.s005.docx]

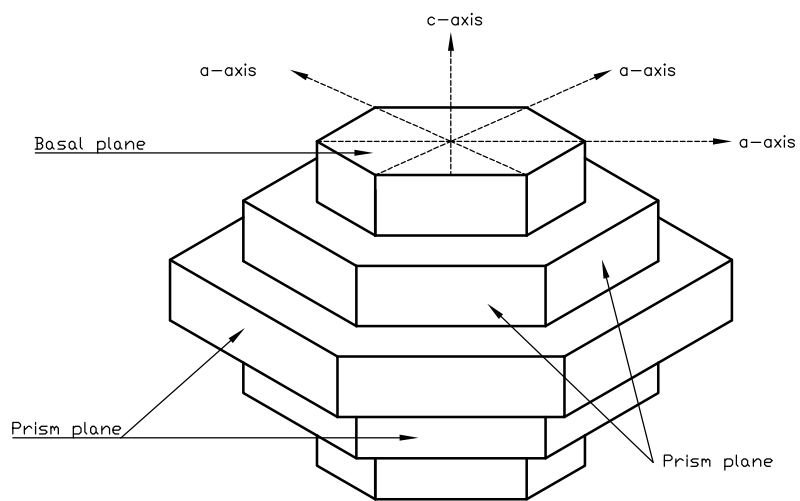

Supplement: S6 Fig — The majority of ice active proteins preferentially bind to the prism planes, inhibiting the growth of ice along these planes and creating hexagonal discs. Continued ice growth on the basal planes and continued binding to prism faces results in the formation of truncated bipyrimidal ice crystals. Figure adapted from Davies and Hew [25]. (PDF) [file pone.0116084.s006.pdf]

Control

Frozen

*P. davidi*

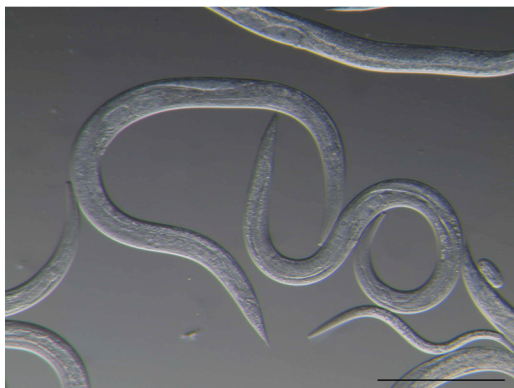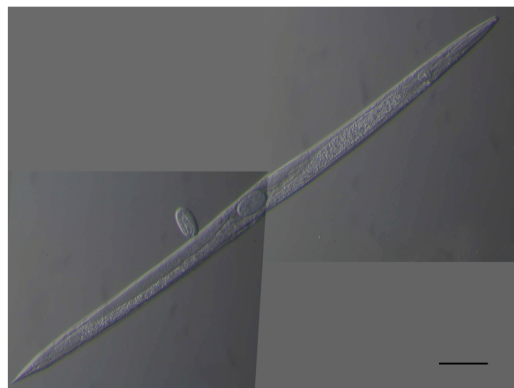

*P. superbis*

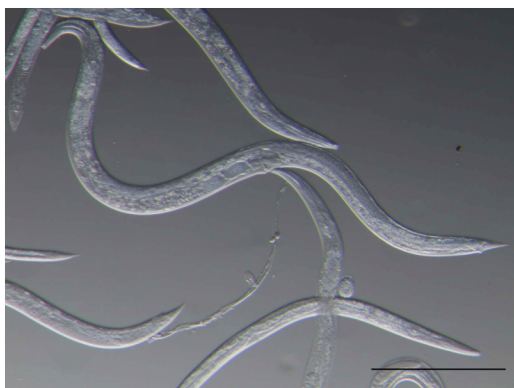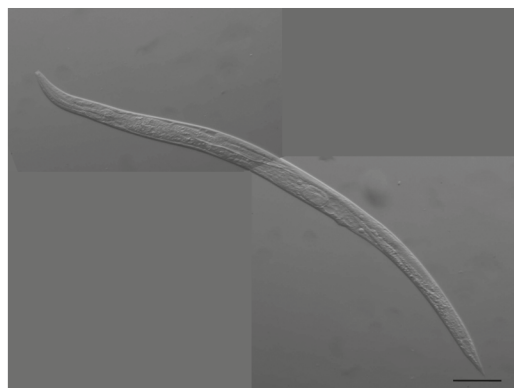

*P. paetzoldi*

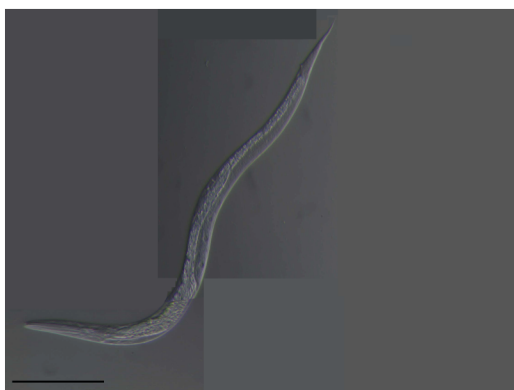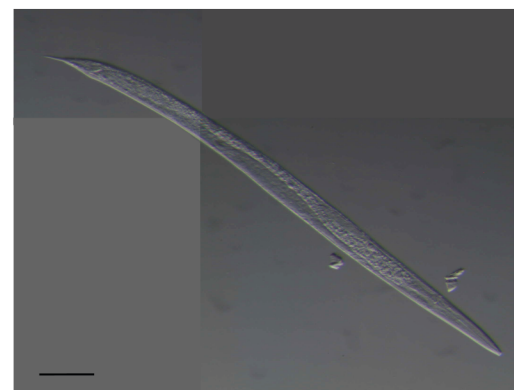

JU1646

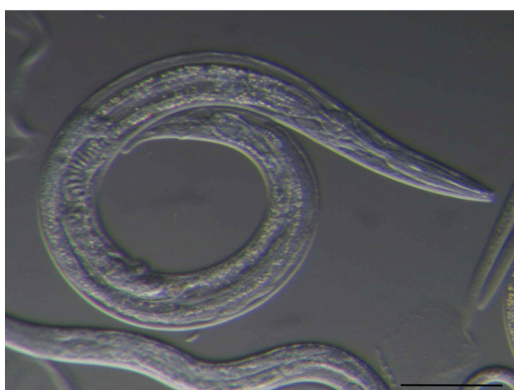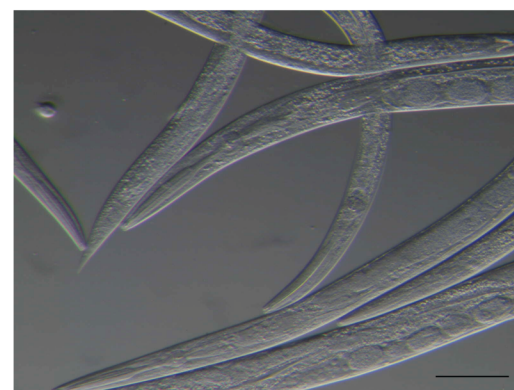

Supplement: S7 Fig — The nematodes are: Panagrolaimus davidi (Antarctica) and P. superbus (Surtsey, Iceland), both freezing tolerant species; Panagrolaimus sp. JU1646 (Cape Verde), a strain with weak freezing tolerance and P. paetzoldi (Netherlands), a freezing sensitive species. Scale bar: strains a, c and g, 500 μm; strains b, d, e, f and h, 200 μm. (PDF) [file pone.0116084.s007.pdf]
