# Supplementary material for: Anhydrobiosis and Freezing-Tolerance: Adaptations That Facilitate the Establishment of Panagrolaimus Nematodes in Polar Habitats
Source: PLoS One. 2015 Mar 6;10(3):e0116084. doi: 10.1371/journal.pone.0116084 (PMC4352009; doi:10.1371/journal.pone.0116084)
Supplement: S1 Table — (DOCX) [file pone.0116084.s008.docx]

**Table S1**. Source of the *Panagrolaimus* isolates used in this study. The names of newly isolated strain are indicated in bold underlined font.

**Species and Strain Geographical Location Habitat Source Reproduction**

*Panagrolaimus* *davidi* (CB1) Ross Island McMurdo Sound, Moss and algae^1^ Prof. David Wharton Parthenogenetic

Antartica^1^

*Panagrolaimus paetzoldi* Paulina saltmarsh, Westerscheldt Decomposing cordgrass (*Spartina anglica*) ^2^ Dr. Ilse De Mesel Male/female^3^

Estuary, Netherlands^2^

*Panagrolaimus* *rigidus* (AF36) Fayette County, Soil CGC^5^ Male/female

Pennsylvania, USA^4^ (isolated by Prof. Andras Fodor)

*Panagrolaimus superbus* (DF5050) Surtsey Island, Iceland^6^ Gull’s nest in a lava cavity. Prof. Bjorn Sohlenius Male/female

(Nest primarily made from moss

*Rhacomitrium* sp.)^6^

***Panagrolaimus* sp. AS01** Leixlip, Co. Kildare, Ireland A roof gutter Dr. Adam Shannon Male/female

***Panagrolaimus* sp. AS03** Maynooth, Co. Kildare, Ireland Roof moss (*Bryum capillare* Hedw) Dr. Adam Shannon Male/female

*Panagrolaimus* sp. JB115 Forest Falls, San Bernadino Pine forest soil^7^ Prof Steven Nadler Parthenogenetic^3^ County, California^7^ (isolated by Doug Edelman)

*Panagrolaimus* sp. JB051 Diourbel, central Senegal^7^ Soil Prof Steven Nadler Parthenogenetic^3^

(isolated by Dr. Pierre Baujard)

*Panagrolaimus* sp. JU765 Yangshuo, Guangxi, China^8^ Soil at edge of rice paddy Isolated by Dr. M.-A. Félix Hermaphrodite^8^

***Panagrolaimus* sp. JU1361** Periyar Natural Preserve, A rotting orange-colored fruit Isolated by Dr. M.-A. Félix Male/female^8^

Kerala, India^8^

***Panagrolaimus* sp. JU1365** Between Periyar and Madurai, A rotting Okra pod Isolated by Dr. M.-A. Félix Male/female^8^

Tamil Nadu, India^8^

***Panagrolaimus* sp. JU1366** Between Madurai and Tanjore, A rotting cactus stem Isolated by Dr. M.-A. Félix Male/female^8^

Tamil Nadu, India^8^

***Panagrolaimus* sp. JU1367** Between Madurai and Tanjore, Soil Isolated by Dr. M.-A. Félix Male/female^8^

Tamil Nadu, India^8^

***Panagrolaimus* sp. JU1369** Cuddatore, Tamil Nadu, India^8^ A mix of rotting fruits, soil and coconut debris Isolated by Dr. M.-A. Félix Male/female^8^

***Panagrolaimus* sp. JU1371** Government Place, Pondicherry, Soil and leaf litter Isolated by Dr. M.-A. Félix Male/female^8^

India^8^

***Panagrolaimus* sp. JU1387** La Réunion, Indian Ocean^8^ Rotting velvet apples (*Diospyros philippensis*) Isolated by Dr. M.-A. Félix Male/female^8^

***Panagrolaimus* sp. JU1645** Between Matinho and Caibros Rotting agave leaves Isolated by Dr. M.-A. Félix Male/female^8^

on Santo Antao Island,

Cape Verde^8^

***Panagrolaimus* sp. JU1646** Ribeira Cumba Small rotting fruits Isolated by Dr. M.-A. Félix Male/female^8^

Santiago Island, Cape Verde^8^

*Panagrolaimus* sp. PS443 Byurakan, Armenia^9^ Soil CGC Male/female

(isolated by Raffi Aroian)

*Panagrolaimus* sp. PS1159 North Carolina, USA Soil CGC Parthenogenetic^3^

(isolated by J. Millar)

*Panagrolaimus* sp. PS1579 Huntington Botanical Gardens Soil CGC Parthenogenetic

San Marino, California, USA (isolated by Dr. M.-A. Félix)

*Panagrolaimus* sp. PS5056 Phnom Phen, Cambodia^10^ John DeModena Male/female

***Panagrolaimus* sp. PS6470** Galileo Hill, Mid Mojave Desert, Soil Isolated by John DeModena Male/female

Kern County, California^10^

*Panagrolaimus* sp. SN103 Eagle Lake Field Station^11^ Tree stump with frass and fungus^11^ Prof Steven Nadler Parthenogenetic^3^

Lassen County, California

- - - 1. Wharton, D.A and Browne, I. M. (1989). A survey of terrestrial nematodes from the McMurdo Sound region, Antarctica. NZ J. Zool. 16, 629-641.
      2. De Mesel. I., Derycke, S., Swings, J., Vincx, M. and Moens, T. (2003). Influence of bacterivorous nematodes on the decomposition of cordgrass. J Exp. Mar. Biol. Ecol. 296, 227-242.
      3. Lewis S.C., Dyal L.A., Hilburn C.F., Weitz S., Liau W.S., LaMunyon C.W., Denver D.R. 2009 Molecular evolution in *Panagrolaimus* nematodes*:* origins of parthenogenesis, hermaphroditism and the Antarctic species *P. davidi*. *BMC Evol Biol* 9, Artn 15. (doi:10.1186/1471-2148-9-15).
      4. Prof. Andras Fodor, Department of Entomology, Ohio State University, OARDC, Wooster, OH, personal communication.
      5. Caenorhabditis Genetics Center, University of Minnesota, Minneapolis, MN 55455, USA.
      6. Bostrom, S. (1998). Descriptions and morphological variability of three populations of *Panagrolaimus* Fuch 1930 (Nematoda: Panagrolaimidae). *Nematologica* 34, 144-155.
      7. Dr. Manuel Mundo-Ocampo, Department of Nematology, University of California Riverside, personal communication
      8. The Félix Laboratory Strain Database, http://www.justbio.com/worms/.
      9. Aroian, R. V., Carta, L., Kaloshian, I. and Sternberg, P. W. (1993). A free-living *Panagrolaimus* sp. from Armenia can survive in anhydrobiosis for 8.7 years. *J. Nematol.* 25, 500-502.
      10. John DeModena, Sternberg Lab., Division of Biology 156-29, California Institute of Biology, Pasadena, Ca 91125, USA, personal commumication.
      11. Prof. Steven Nadler, Department of Nematology, University of California Davis, personal communication.
