# Supplementary material for: Anhydrobiosis and Freezing-Tolerance: Adaptations That Facilitate the Establishment of Panagrolaimus Nematodes in Polar Habitats
Source: PLoS One. 2015 Mar 6;10(3):e0116084. doi: 10.1371/journal.pone.0116084 (PMC4352009; doi:10.1371/journal.pone.0116084)
Supplement: S2 Table — (DOCX) [file pone.0116084.s009.docx]

**Table S2.** Principal component analysis of the anhydrobiotic and freezing-tolerance phenotypes of *Panagrolaimus* species and strains.

|  | **PC axis** | | | | | | |
| --- | --- | --- | --- | --- | --- | --- | --- |
|  | **1** | **2** | **3** | **4** | **5** | **6** | **7** |
| Eigenvalues | 4.01 | 1.74 | 0.65 | 0.27 | 0.21 | 0.09 | 0.03 |
| Explained proportion of variation (%) | 57.34% | 24.86% | 9.33% | 3.91% | 2.94% | 1.24% | 0.34% |
| Cumulative proportion of variation (%) | 57.34% | 82.20% | 91.53% | 95.44% | 98.38% | 99.62% | 100% |
| **Treatment** | **Eigenvectors** | | | | | | |
| Unacclimated | -0.32 | -0.50 | 0.20 | 0.00 | 0.75 | 0.10 | -0.20 |
| Acclimated | -0.26 | -0.54 | 0.45 | -0.28 | -0.58 | -0.12 | 0.10 |
| 0 hours preconditioning | -0.28 | -0.44 | -0.64 | 0.51 | -0.22 | 0.11 | 0.05 |
| 24 hours preconditioning | -0.43 | 0.10 | -0.40 | -0.70 | 0.08 | 0.35 | 0.14 |
| 48 hours preconditioning | -0.43 | 0.31 | -0.17 | 0.04 | -0.14 | -0.69 | -0.43 |
| 72 hours preconditioning | -0.44 | 0.29 | 0.21 | 0.28 | 0.12 | -0.15 | 0.75 |
| 96 hours preconditioning | -0.43 | 0.28 | 0.34 | 0.30 | -0.13 | 0.58 | -0.42 |
