# Supplementary material for: Anhydrobiosis and Freezing-Tolerance: Adaptations That Facilitate the Establishment of Panagrolaimus Nematodes in Polar Habitats
Source: PLoS One. 2015 Mar 6;10(3):e0116084. doi: 10.1371/journal.pone.0116084 (PMC4352009; doi:10.1371/journal.pone.0116084)
Supplement: S3 Table — (DOCX) [file pone.0116084.s010.docx]

**Table S3.** **GenBank accession numbers for the 28S rDNA D3 region sequences used in *Panagrolaimus* phylogeny reconstructions [Fig. 1 (a) and Fig. S3].**

**Species Accession Number**

*Panagrolaimus* *davidi* AY878385

*Panagrolaimus paetzoldi* FJ717478

*Panagrolaimus* *rigidus* (AF36) AY878379

*Panagrolaimus superbus* AY878376  *Panagrolaimus* sp. AS01 FJ717472

*Panagrolaimus* sp. AS03 FJ717474

*Panagrolaimus* sp. JB051 FJ717476

*Panagrolaimus* sp. JB115 KC522693

*Panagrolaimus* sp. JU765 KC522694

*Panagrolaimus* sp. JU 1361 KC522695

*Panagrolaimus* sp. JU 1365 KC522696

*Panagrolaimus* sp. JU 1366 KC522697

*Panagrolaimus* sp. JU 1367 KC522698

*Panagrolaimus* sp. JU 1369 KC522699

*Panagrolaimus* sp. JU 1371 KC522700

*Panagrolaimus* sp. JU 1387 KC522701

*Panagrolaimus* sp. JU 1645 KC522702

*Panagrolaimus* sp. JU 1646 KC522703

*Panagrolaimus* sp. PS443 AY878381

*Panagrolaimus* sp. PS1159 AY878382

*Panagrolaimus* sp. PS5056 KC522704

*Panagrolaimus* sp. PS1579 AY878383

*Panagrolaimus* sp. PS6470 KC522705

*Panagrolaimus* sp. SN103 FJ717477

|  |
| --- |
